# Mathematical modeling and nonlinear bilateral multivalued stochastic integral equations

**DOI:** 10.1371/journal.pone.0323411

**Published:** 2025-06-04

**Authors:** Marek T. Malinowski

**Affiliations:** Department of Applied Mathematics, Tadeusz Kościuszko Cracow University of Technology, Kraków, Poland; Sefako Makgatho Health Sciences University Faculty of Health Sciences, SOUTH AFRICA

## Abstract

In this paper, we begin our study by exploring a hypothetical model of stochastic growth of a population, using a single-valued stochastic integral equation that incorporates the control of feeding and harvest. Taking into account the inaccuracies and uncertainties in the measurements, we are led to a nonlinear bilateral multivalued stochastic integral equation that contains multivalued stochastic integrals on both sides of the equation. Due to the possibility of absence of an element opposite to a fixed set, such an equation cannot be reduced to classical unilateral notation with the sign of sum of sets only on one side. The fundamental question arises: Is there a solution to the equation under consideration, and is it the only one? By imposing on the coefficients of the equation the condition of satisfying a certain integral inequality, we prove the existence and uniqueness of solution of the considered equation. The result is preceded by a few lemmas with the sequence of approximate solutions. We also show that solutions have the property of stability. Finally, it has been demonstrated that the results obtained can be applied to establish corresponding theorems for deterministic bilateral multivalued integral equations.

## Introduction

Multivalued differential equations were introduced in [1] as extensions of ordinary differential equations. These equations have researchers who continually expand this research domain. Notable achievements in this field include: existence of solutions [2], asymptotic and strict stability [3,4], random dynamics [5], equations with causal maps [6,7], small perturbations [8], solution established by multivalued fixed point theorem [9,10], variation of the constants formula [11], periodic solutions [12], equations on time scales [13], equations with second type Hukuhara derivative [14,15], symmetric functional equations [16], numerical solution [17], relaxation [18], fractional derivative [19], exponential stability [20], order of convergence [21], best proximity points [22], multivalued operators [23]. In these equations, there appear multivalued mappings, known as multifunctions, and the derivative may also be multivalued. These equations gained prominence because of their ability to more accurately depict the dynamics of phenomena within environments characterized by uncertain data. For instance, measurement inaccuracies can result in initial values being represented as intervals (i.e., sets of multiple values) rather than precise single numerical values. Furthermore, the rate of changes in the studied phenomenon may assume various values. Consequently, multivalued differential equations have emerged as mathematical models that consider such properties. Currently, this has become a distinct area of scientific research with its own analytical methods [24–27], using either the inclusion sign or the equality sign in the relation/equation considered. Furthermore, there has been an effort to extend the investigation of deterministic multivalued differential equations to a stochastic version, enabling the modeling of unpredictable phenomena, for example [28–37]. However, in the mentioned literature, three various methods of constructing multivalued stochastic equations can be found. The first one [28,29] involves considering inclusions instead of equations, and thus the solutions are single-valued mappings. In the second method [30–34], genuinely multivalued equations are considered and the solutions are multivalued stochastic processes, but due to certain limitations, the necessary Itô-type stochastic integral for such equations is single-valued and not multivalued. The reason for this approach is the conclusions drawn in papers [39] and [40], namely, that the method of constructing the multivalued Itô integral so that it is a multivalued random variable leads to the possibility that this integral takes on values that are unbounded sets, rendering it useless for applying to stochastic equations and modeling any phenomena. In the subsequent method, utilized in [35–37], the Itô stochastic integral is not a multivalued random variable, but a subset of the infinite-dimensional space of square-integrable random variables. This approach allows for the consideration of equations with a multivalued Itô-type integral. However, the solutions are multivalued mappings with values in the hyperspace of subsets of the space of random variables. These solutions are of a different kind than those in the previous method (the values of solution are not real numbers) and unfortunately, this makes it impossible to simulate such solutions on a computer. However, this limitation may pose certain challenges and may trigger attempts to overcome it in future research. The last third method of constructing multivalued stochastic equations is adopted in the current paper. We leave the study of these equations in the theoretical realm. Although, as we show in this paper, the emergence of such equations may stem from attempts to describe practical issues, such as population growth. To announce the research presented here and emphasize the resulting benefits, we will introduce a brief overview of the essence of the proposed material.

In this paper, to make the abstract concept tangible, we introduce a hypothetical scenario involving a fish population in a controlled pond. The fish density in the pond depends on stochastic factors (e.g., environmental randomness) and is initially modeled using a single-valued stochastic equation. However, as the model incorporates ambiguity - such as uncertain initial population or environmental conditions - the description evolves into a multivalued stochastic equation. This transition demonstrates how considering additional uncertainties enriches the model. So, led from a hypothetical example of modeling a population growth by single-valued stochastic differential equation, we proceed and analyze bilateral multivalued stochastic differential equations in their integral form

$$\;U(t)+(M)\int_{0}^{t}A_{1}(s,U(s))ds+(M)\int_{0}^{t}A_{2}(s,U(s))d\tilde{W}(s)\mathrm{\backslash nonumber}\;=U_{0}+(M)\int_{0}^{t}A_{3}(s,U(s))ds+(M)\int_{0}^{t}A_{4}(s,U(s))dW(s),$$ (1)

where *t* can be seen as a time variable, *U*_0_ represents a collection of potential initial random variables, $A_{1},A_{2},A_{3},A_{4}$ indicate multivalued stochastic processes, and $W,\tilde{W}$ are two real-valued Wiener processes (which may not be independent). It can be noted that multivalued stochastic integral equations generalize single-valued equations by accounting for stochasticity and additional ambiguity. They represent situations where multiple potential outcomes could arise under the same conditions due to the interplay of stochastic and ambiguous uncertainties. This generalization helps capture the complexity of systems more comprehensively. In the Eq (1), all integrals are multivalued and this is symbolized by (*M*) before the integral sign. The bilateral form of the equation is unique and properly justified for equations considering modeling under conditions of imprecision or ambiguity. It is generally irreducible to a notation with single Lebesgue and Itô integrals on one side. This unilateral notation can only be referred to in the case of equations with single-valued mappings. Moreover, such multivalued bilateral equations cover the entire range of properties of their solutions in relation to the behavior of the function t↦diam U(t), where diamV denotes the diameter of the set *V*. For solutions *U* of (1), it can be nondecreasing, nonincreasing, constant, or even nonmonotonic. They also cover some particular form of equations studied previously in the literature, because the form of the Eq (1) under consideration contains two main particular representations. The first form, which is a natural extension of classical single-valued stochastic differential equations, is given by

$$U(t)=U_{0}+(M)\int_{0}^{t}A_{3}(s,U(s))ds+(M)\int_{0}^{t}A_{4}(s,U(s))dW(s).$$ (2)

Let us mention that these equations and their form have a certain property that can be seen as a drawback. That is, every solution *U* of the Eq (2) satisfies: the function t↦diam U(t) is nondecreasing (Theorem 3.1 [35]). This means that these equations are appropriate only in the case of increasing impreciseness (or nondecreasing, at least) over time. To overcome the indicated barrier, a second particular case of the Eq (1) has been introduced, namely

$$U(t)+(M)\int_{0}^{t}A_{1}(s,U(s))ds+(M)\int_{0}^{t}A_{2}(s,U(s))d\tilde{W}(s)=U_{0},$$ (3)

which, although less intuitive, highlights the intricacies and unique features of multivalued analysis. Now, the solutions of the equation satisfy (Theorem 3.2 [35]): the function t↦diam U(t) is nonincreasing. Thus, these equations are suitable for situations where a decrease in uncertainty is expected. Hence, equations of the form (3) are not equivalent to those of the form (2), even if $A_{3}=(-1)\cdot A_{1}$ and $A_{4}=(-1)\cdot A_{2}$. This is formally because the hyperspace of subsets of a fixed set lacks a linear space structure. There are significant issues with the existence of inverse elements and the subtraction of sets. As a result, Eq (3) cannot generally be transformed into Eq (2), except when all mappings and data are single-valued. To obtain solutions where the function t↦diam U(t) changes its monotonicity, we proposed examining the bilateral multivalued Eq (1) and this has been done in two papers so far, [36] and [37]. These equations are the focus of this paper. Our objective is to demonstrate that bilateral multivalued stochastic integral Eq (1) have unique solutions under certain conditions different from the standard Lipschitz condition used in [36] and a weakened Lipschitz condition in [37]. We will require the drift and diffusion coefficients in the equation to meet a specific integral inequality. This condition is more general than the two mentioned in [36,37]. The method of obtaining the solution involves using a sequence of approximations, which we show to be well defined, uniformly jointly bounded, and satisfying the Cauchy condition. Then we demonstrate that its limit is a solution of Eq (1). We do not use the fixed-point method if only because its use would require more restrictive assumptions about the existence of the Hukuhara differences, thereby making the resulting outcome weaker compared to the one presented in this paper. We will show that the solutions continuously depend on the equation data, confirming the stability of the solutions for the considered equations. This property is desirable because it means that small changes in the equation’s data lead to only small changes in the solution. In the final part of the paper, we address the study of deterministic bilateral multivalued integral equations with values in subsets of the real line. We indicate that by proceeding as in the previous proofs one can obtain results for deterministic equations, which constitute a separate branch of multivalued analysis. Moreover, in this case, it was possible to demonstrate that the restrictive assumption of the existence of certain Hukuhara differences of subsets of ℝ can be completely omitted.

## Preliminaries

In this section, we summarize various concepts and facts of the multivalued analysis, which will be beneficial in our subsequent discussions. These details can be found in sources such as [37], but we provide them here for the reader’s convenience.

Consider $\left(𝒳,\|\cdot {\|}_{𝒳}\right)$ as a separable Banach space. The notation ${𝒫}_{c}^{b}(𝒳)$ represents the collection of all non-empty, closed, bounded, and convex subsets of 𝒳. In ${𝒫}_{c}^{b}(𝒳)$, we utilize the Pompeiu–Hausdorff metric ${𝒟}_{𝒳}$, defined as

$${𝒟}_{𝒳}(U,V)=\max\left\{\underset{u\in U}{\sup}\underset{v\in V}{\inf}\|u-v{\|}_{𝒳},\underset{v\in V}{\sup}\underset{u\in U}{\inf}\|u-v{\|}_{𝒳}\right\}.$$ (4)

According to [25], the metric space $\left({𝒫}_{c}^{b}(𝒳),{𝒟}_{𝒳}\right)$ is complete. If one considers the family of nonempty, closed, and convex subsets of a separable and reflexive Banach space 𝒳 with the Mosco topology $\tau_{M_{𝒳}}$, then it forms a Polish topological space. The Mosco topology is metrizable and weaker than the topology $\tau_{{𝒟}_{𝒳}}$ induced by the Pompeiu–Hausdorff metric ${𝒟}_{𝒳}$.

Addition for $U,V\in {𝒫}_{c}^{b}(𝒳)$ follows Minkowski’s definition:


U+V={u+v:u∈U,v∈V},

and multiplication by a scalar r∈ℝ is defined as


rU={ru:u∈U}.

In this paper, the difference of sets $U,V\in {𝒫}_{c}^{b}(𝒳)$ is interpreted in the sense of Hukuhara, such that the difference $U⊖V\in {𝒫}_{c}^{b}(𝒳)$ is the set satisfying


(U⊖V)+V=U.

Note that the set U⊖V may not always exist, and there is no simple criterion for the existence of the Hukuhara difference.

In the following sections, we will use the properties: for $U,V,W,X\in {𝒫}_{c}^{b}(𝒳)$

(P1) if U⊖V exists, then ${𝒟}_{𝒳}(U⊖V,\{\theta_{𝒳}\})={𝒟}_{𝒳}(U,V)$, where $\theta_{𝒳}$ denotes the zero element in 𝒳,
(P2) ${𝒟}_{𝒳}(U+V,W+X)⩽{𝒟}_{𝒳}(U,W)+{𝒟}_{𝒳}(V,X)$,
(P3) ${𝒟}_{𝒳}(U+W,V+W)={𝒟}_{𝒳}(U,V)$,
(P4) if U⊖V and W⊖X exist, then ${𝒟}_{𝒳}(U⊖V,W⊖X)⩽{𝒟}_{𝒳}(U,W)+{𝒟}_{𝒳}(V,X)$.

Next, we discuss fundamental facts about measurable multifunctions. Let (E,ℰ,μ) be a measure space. A multivalued mapping $F:E\to {𝒫}_{c}^{b}(𝒳)$ is called ℰ-measurable if:


{e∈E:F(e)∩O≠∅}∈ℰfor every open setO⊂𝒳.

A measurable multifunction *F* is ${\text{L}}^{\text{p}}$-integrally bounded (p⩾1) if $e\mapsto {𝒟}_{𝒳}(F(e),\{\theta_{𝒳}\})$ belongs to $L^{p}(E,ℰ,\mu ;\mathbb{R}).$

Define I:=[0,T], where T<∞, and denote the Borel σ-algebra of subsets of *I* by $\beta_{I}$. Let $\left(\Omega ,𝒜,\{{𝒜}_{t}{\}}_{t\in I},P\right)$ be a complete filtered probability space satisfying the usual hypotheses, i.e., $\{{𝒜}_{t}{\}}_{t\in I}$ is an increasing and right-continuous family of sub-σ-algebras of 𝒜 and ${𝒜}_{0}$ contains all *P*-null sets. By 𝒩 we denote the σ-algebra of non-anticipating elements in I×Ω, i.e.,


$$𝒩=\{A\in \beta_{I}⊗𝒜:A^{t}\in {𝒜}_{t}\;\text{for every}\;t\in I\},$$

where $A^{t}=\{\omega :(t,\omega )\in A\}$. By λ we denote the Lebesgue measure in $\left(I,\beta_{I}\right)$.

Consider the space


$$L_{𝒩}^{2}(\lambda \times P):=L^{2}(I\times \Omega ,𝒩,\lambda \times P;{\mathbb{R}}^{d}).$$

For every $u\in L_{𝒩}^{2}(\lambda \times P)$ and τ,t∈I, τ<t, the Itô stochastic integral $\int_{\tau }^{t}u(s)dW(s)$ exists, and $\int_{\tau }^{t}u(s)dW(s)\in L^{2}(\Omega ,{𝒜}_{t},P;{\mathbb{R}}^{d})\subset L^{2}(\Omega ,𝒜,P;{\mathbb{R}}^{d})$, [38].

Let $U:I\times \Omega \to {𝒫}_{c}^{b}({\mathbb{R}}^{d})$ be a multivalued stochastic process, i.e., a family of 𝒜-measurable multivalued mappings $U(t,\cdot ):\Omega \to {𝒫}_{c}^{b}({\mathbb{R}}^{d})$ for t∈I. We call *U* non-anticipating if U(·,·) is an 𝒩-measurable multivalued mapping. Define the set

$$S_{𝒩}^{2}(U,\lambda \times P):=\{u\in L_{𝒩}^{2}(\lambda \times P):u\in U,\;\lambda \times P\text{-a.e.}\}.$$ (5)

If $U:I\times \Omega \to {𝒫}_{c}^{b}({\mathbb{R}}^{d})$ is non-anticipating and $L_{𝒩}^{2}(\lambda \times P)$-integrally bounded, then by the Kuratowski and Ryll-Nardzewski Selection Theorem (see [41]), it follows that $S_{𝒩}^{2}(U,\lambda \times P)\neq \emptyset$. For such a multivalued stochastic process *U*, we can define the multivalued stochastic Itô integral with respect to the $\left\{{𝒜}_{t}\right\}$-Wiener process $\{W(t){\}}_{t\in I}$. Specifically, for τ,t∈I, τ<t, this integral represents the set


$$(M)\int_{\tau }^{t}U(s)dW(s):=\left\{\int_{\tau }^{t}u(s)dW(s):u\in S_{𝒩}^{2}(U,\lambda \times P)\right\}.$$

Thus, $\left(M)\int_{\tau }^{t}U(s)dW(s)\subset L^{2}(\Omega ,{𝒜}_{t},P;{\mathbb{R}}^{d})\subset L^{2}(\Omega ,𝒜,P;{\mathbb{R}}^{d}\right)$. Similarly, for the multivalued stochastic process *U*, we can define the multivalued stochastic Aumann integral, denoted by $(M)\int_{\tau }^{t}U(s)ds$:


$$(M)\int_{\tau }^{t}U(s)ds:=\left\{\int_{\tau }^{t}u(s)ds:u\in S_{𝒩}^{2}(U,\lambda \times P)\right\}.$$

Naturally, $(M)\int_{\tau }^{t}U(s)ds\subset L^{2}(\Omega ,{𝒜}_{t},P;{\mathbb{R}}^{d})\subset L^{2}(\Omega ,𝒜,P;{\mathbb{R}}^{d}).$

The following properties of stochastic integrals (see, e.g. [37]) are instrumental in analyzing multivalued stochastic integral equations.

**Lemma 1.** *Consider $U,V:I\times \Omega \to {𝒫}_{c}^{b}({\mathbb{R}}^{d})$ as nonanticipating multivalued stochastic processes that are integrally bounded in the $L_{𝒩}^{2}(\lambda \times P)$ sense. Let τ,t∈I with τ<t. Then*

*(i)*
  $${𝒟}_{L^{2}}^{2}\left((M)\int_{\tau }^{t}U(s)dW(s),(M)\int_{\tau }^{t}V(s)dW(s)\right)⩽\int_{[\tau ,t]\times \Omega }{𝒟}_{{\mathbb{R}}^{d}}^{2}(U,V)ds\times dP$$
*and*
$${𝒟}_{L^{2}}^{2}\left((M)\int_{\tau }^{t}U(s)ds,(M)\int_{\tau }^{t}V(s)ds\right)⩽(t-\tau )\int_{[\tau ,t]\times \Omega }{𝒟}_{{\mathbb{R}}^{d}}^{2}(U,V)ds\times dP,$$
*(ii) for a∈[τ,t]*
$$\left(M)\int_{\tau }^{t}U(s)dW(s)=(M)\int_{\tau }^{a}U(s)dW(s)+(M)\int_{a}^{t}U(s)dW(s\right)$$
*and*
$$(M)\int_{\tau }^{t}U(s)ds=(M)\int_{\tau }^{a}U(s)ds+(M)\int_{a}^{t}U(s)ds),$$
*(iii) the mappings*
$$(M)\int_{\tau }^{t}U(s)ds=(M)\int_{\tau }^{a}U(s)ds+(M)\int_{a}^{t}U(s)ds),$$
$$(M)\int_{\tau }^{t}U(s)ds=(M)\int_{\tau }^{a}U(s)ds+(M)\int_{a}^{t}U(s)ds),$$
*are ${𝒟}_{L^{2}}$-continuous.*

Henceforth, the space $L^{2}(\Omega ,𝒜,P;{\mathbb{R}}^{d})$ will be referred to as ${\text{L}}^{2}$, while $L^{2}(\Omega ,{𝒜}_{t},P;{\mathbb{R}}^{d})$ for t∈I will be denoted as $L_{t}^{2}$. We assume that the σ-algebras 𝒜 and ${𝒜}_{t}$ are separable with respect to the probability measure *P*. Consequently, the spaces ${\text{L}}^{2}$ and $L_{t}^{2}$, endowed with their usual norms, are separable.

## Modeling leading to nonlinear bilateral multivalued stochastic integral equations

In this part, we will show how a hypothetical problem of dynamics of a population growth, taking into account uncertainty, can lead to equations of the type studied in this paper, that is, to bilateral multivalued stochastic integral equations. We also point out that such equations can be used in modeling optimality problems.

Suppose, purely hypothetically and for the sake of sparking imagination, that a breeder has his own giant artificial breeding pond and he is breeding a certain species of fish (if it would be more convenient, one can think of a different species and not necessarily fish). Let us assume that the density of fish in the pond depends on certain random factors and that the breeder can control the amount of fish by controlling the amount of food fed. If we denote by *u*(*t*) the density of fish in the pond at the moment t∈I, then we can treat it as a random element that can be modeled by the controlled stochastic integral equation, written in a very general form as

$$u(t)=u_{0}+\int_{0}^{t}a(u(s),c(s))ds+\int_{0}^{t}b(u(s),c(s))dW(s),\;\;\;t\in I,\;\;P\text{-a.e.},$$ (6)

where $u_{0}:\Omega \to \mathbb{R}$ denotes initial number of individuals, $a:{\mathbb{R}}^{2}\to \mathbb{R}$ stands for drift coefficient of growth, $b:{\mathbb{R}}^{2}\to \mathbb{R}$ is a volatility coefficient of growth, *c* denotes a strategy of feeding, c∈C, *C* means a set of feeding actions - controls, *W* is the Wiener process. A very simplified version of Eq (6) was used to demonstrate the application of the stochastic equation to model population growth in [38], pages 63–65.

Eq (6) can be extended if we consider the harvest of fish at every time t∈I. Then (6) can be completed to the form


$$u(t)=u_{0}+\int_{0}^{t}a(u(s),c(s),l(s))ds+\int_{0}^{t}b(u(s),c(s),l(s))dW(s)\;-\int_{0}^{t}\tilde{a}(u(s),c(s),l(s))ds-\int_{0}^{t}\tilde{b}(u(s),c(s),l(s))d\tilde{W}(s),\;\;\;t\in I,\;\;P\text{-a.e.},$$

where $\tilde{a}$, $\tilde{b}$, $\tilde{W}$ have similar meaning as before but now apply to the fish harvesting and *l* stands for fish harvesting strategy, *L* is a set of admissible controls *l*. Since this is an equation in the space ${\mathbb{R}}^{1}$, it can be simply rewritten as

$$\;u(t)+\int_{0}^{t}\tilde{a}(u(s),c(s),l(s))ds+\int_{0}^{t}\tilde{b}(u(s),c(s),l(s))d\tilde{W}(s)\;=u_{0}+\int_{0}^{t}a(u(s),c(s),l(s))ds+\int_{0}^{t}b(u(s),c(s),l(s))dW(s),\;\;t\in I,\;\;P\text{-a.e.}$$ (7)

Thinking of *u*(*t*) for t∈I as of a random variable from the space $L^{2,1}=L^{2}(\Omega ,𝒜,P;{\mathbb{R}}^{1})$, we can transform (7) to an equation in the space ${\text{L}}^{2,1}$, i.e. to the equation

$$\;u(t)+\int_{0}^{t}\hat{\tilde{a}}(u(s),c(s),l(s))ds+\int_{0}^{t}\hat{\tilde{b}}(u(s),c(s),l(s))d\tilde{W}(s)\;=u_{0}+\int_{0}^{t}\hat{a}(u(s),c(s),l(s))ds+\int_{0}^{t}\hat{b}(u(s),c(s),l(s))dW(s),\;\;t\in I,$$ (8)

where the mappings $\hat{a},\hat{b},\hat{\tilde{a}},\hat{\tilde{b}}:I\times \Omega \times L^{2,1}\times C\times L\to {\mathbb{R}}^{1}$ are defined as follows


$$\hat{a}(s,\omega ,u,c,l):=a(u(\omega ),c(s,\omega ),l(s,\omega ))\;\;\;\text{and}\;\;\;\hat{b}(s,\omega ,u,c,l):=b(u(\omega ),c(s,\omega ),l(s,\omega )),$$

$$\hat{\tilde{a}}(s,\omega ,u,c,l):=\tilde{a}(u(\omega ),c(s,\omega ),l(s,\omega ))\;\;\;\text{and}\;\;\;\hat{\tilde{b}}(s,\omega ,u,c,l):=\tilde{b}(u(\omega ),c(s,\omega ),l(s,\omega )).$$

These equations describe a phenomenon in which uncertainty arises from stochastic factors, such as random changes or noise. The single-valued equations model situations where the system’s behavior can be predicted as a unique outcome for given inputs, albeit under randomness. However, to use Eq (8) the breeder must precisely determine *u*_0_. This is almost never met, for example, for reasons of errors generated by measuring devices. Hence, in the process of modeling, it can be observed that real-world systems often involve multiple sources of uncertainty. Beyond stochastic randomness, there is ambiguity - another layer of uncertainty that does not have a stochastic origin. Ambiguity could stem from incomplete knowledge about the system. So, using (8) seems to be not adequate enough. Based on the expert knowledge of the breeder, it could be determined, for instance, that *u*_0_ is an ${𝒜}_{0}$-measurable random variable with values bounded by a constant α>0. The uncertainty appearing in the determination of the initial value *u*_0_ leads to the use of a set *U*_0_ of random variables instead of one random variable *u*_0_. Then the initial number of individuals can be viewed as the following set


$$U_{0}:=\{u_{0}\in L_{0}^{2,1}:P(\{\omega :0⩽u_{0}(\omega )⩽\alpha \})=1\}\in {𝒫}_{c}^{b}(L_{0}^{2,1}),$$

where $L_{0}^{2,1}=L^{2}(\Omega ,{𝒜}_{0},P;{\mathbb{R}}^{1})$. Now, the initial value with uncertainty modeled by the set *U*_0_ should be processed giving the dynamics of the fish density in pond in terms of sets *U*(*t*) that should provide an estimate of the true level of fish density *u*(*t*). This dynamics could be described by the bilateral multivalued stochastic integral equation

$$\;U(t)+(M)\int_{0}^{t}A(s,U(s))ds+(M)\int_{0}^{t}B(s,U(s))dW(s)\;=U_{0}+(M)\int_{0}^{t}\tilde{A}(s,U(s))ds+(M)\int_{0}^{t}\tilde{B}(s,U(s))d\tilde{W}(s),$$ (9)

where $A,\tilde{A},B,\tilde{B}:I\times \Omega \times {𝒫}_{c}^{b}(L^{2,1})\to {𝒫}_{c}^{b}({\mathbb{R}}^{1})$ are defined as follows


$$A(s,\omega ,U):=\overset{―}{co}\left(\underset{u\in U}{⋃}\underset{c\in C}{⋃}\underset{l\in L}{⋃}\hat{a}(s,\omega ,u,c,l)\right)$$

$$\text{and}\;\;\;B(s,\omega ,U):=\overset{―}{co}\left(\underset{u\in U}{⋃}\underset{c\in C}{⋃}\underset{l\in L}{⋃}\hat{b}(s,\omega ,u,c,l)\right),$$

$$\tilde{A}(s,\omega ,U):=\overset{―}{co}\left(\underset{u\in U}{⋃}\underset{c\in C}{⋃}\underset{l\in L}{⋃}\hat{\tilde{a}}(s,\omega ,u,c,l)\right)$$

$$\text{and}\;\;\;\tilde{B}(s,\omega ,U):=\overset{―}{co}\left(\underset{u\in U}{⋃}\underset{c\in C}{⋃}\underset{l\in L}{⋃}\hat{\tilde{b}}(s,\omega ,u,c,l)\right).$$

The notation $\overset{―}{co}(V)$ represents the closure of the convex hull of the set *V*.

Let us point out that the integrals on the left-hand side of the Eq (9) are connected with the fish harvesting, so it can be said that their impact is to decrease the level of uncertainty measured by diameter of the set *U*(*t*), where *U*(*t*) is a set that approximates the true value *u*(*t*). It may be intuitively obvious that the fewer fish in the pond, the lower the uncertainty of the current density assessment, i.e. the grower should be able to assess the density with more accuracy in this case. On the other hand, the integrals on the right-hand side of (9) are responsible for the dynamics of population growth, and hence they increase diameter of the set *U*(*t*). As can be observed, Eq (9) covers both the properties of population decay and population growth.

Having Eq (9), the natural question becomes the existence of its solution. This is one of the main goals of this paper. The multivalued solution could represent information on an approximate dynamics of the fish density. Until now, such equations have been investigated with the assumption that the equation coefficients meet the Lipschitz condition [36] and a slightly more general condition presented in [37]. In this paper, we would like to present the existence of a solution when the Lipschitz condition is weakened even more than in [37]. This is also the natural direction for expanding research of this type of multivalued equations.

Looking at Eqs (8) and (9) once again, it can easily be concluded that between the solutions of both equations there exists a relation expressed by the inclusion u(t)∈U(t) for t∈I, which means that *u* is a continuous selection of multivalued mapping *U*. With each choice of *u*, which is the selection of the solution *U*, one can associate the cost *K*(*u*) borne by the breeder related to achieving the fish density described by *u*. Next, we can ask about the existence of a continuous selection $u^{⋆}$ that realizes the lowest value of the cost. More specifically, one may look for the continuous selection $u^{⋆}$ of the solution *U* to (9) with a property


$$K(u^{⋆})=\inf_{u}\;K(u),$$

where *u* stands for any continuous selection of the solution *U* to the Eq (9). We see again that the issue of the existence of a unique solution to bilateral multivalued stochastic integral Eq (9) is crucial and fundamental. We will therefore address this issue in the next section and some theoretical examinations will be presented. Let us recall that the imaginative example of the fish population has served only as a tool to guide the reader through the conceptual leap from single-valued to multivalued equations. It has shown how ambiguity can arise naturally in modeling and why addressing it leads to more robust representations of reality. Hence, the broader implication is that these multivalued equations are valuable for understanding and predicting the behavior of complex, uncertain systems.

## Theoretical results on nonlinear bilateral multivalued stochastic integral equations

Let us remind that the subject of our research is bilateral multivalued stochastic integral equation

$$\;U(t)+(M)\int_{0}^{t}A_{1}(s,U(s))ds+(M)\int_{0}^{t}A_{2}(s,U(s))d\tilde{W}(s)\;=U_{0}+(M)\int_{0}^{t}A_{3}(s,U(s))ds+(M)\int_{0}^{t}A_{4}(s,U(s))dW(s),$$ (10)

for t∈I, where $U_{0}\in {𝒫}_{c}^{b}(L_{0}^{2})$ is the initial set of ${𝒜}_{0}$-measurable and square integrable random variables, $A_{1},A_{2},A_{3},A_{4}:I\times \Omega \times {𝒫}_{c}^{b}(L^{2})\to {𝒫}_{c}^{b}({\mathbb{R}}^{d})$ are the drift and diffusion coefficients of the equation, *W* and $\tilde{W}$ are real-valued Wiener processes (not necessarily independent), and the symbol (*M*) next to integrals indicates multivalued integrals.

At this point, we will give what we mean by the solution of the equation under consideration. We mention that in the case of Eq (10) it is necessary to think about local rather than global solutions, which is clearly visible in the upcoming condition (A4). Therefore, we introduce the interval J⊂I such that $J=[0,\tilde{T}]$, where $\tilde{T}⩽T$.

**Definition 1.** *A multivalued mapping $U:J\to {𝒫}_{c}^{b}(L^{2})$ is considered a solution to* Eq (10) *if it is ${𝒟}_{L^{2}}$-continuous and meets the condition of* Eq (10) *for every t∈J.*

Obviously, if $\tilde{T}=T$, then the solution *U* is global, but in the case $\tilde{T}<T$ we have a local solution at our disposal.

**Definition 2.** *A mapping $U:J\to {𝒫}_{c}^{b}(L^{2})$ is considered a unique solution to* Eq (10) *if U(t)=V(t) for every t∈J, where V is any other solution (defined on the interval J).*

In the following, we will outline conditions that are broader than those presented in [37] and will enable us to achieve the desired results concerning the existence and uniqueness of the solution to the equation in question. These conditions are as follows:

(A0) $U_{0}\in {𝒫}_{c}^{b}(L_{0}^{2})$,
(A1) the multivalued mappings $A_{1},A_{2},A_{3},A_{4}:I\times \Omega \times {𝒫}_{c}^{b}(L^{2})\to {𝒫}_{c}^{b}({\mathbb{R}}^{d})$ are $𝒩⊗\beta (\tau_{M_{L^{2}}})$-measurable, where $\beta (\tau_{M_{L^{2}}})$ represents the Borel σ-algebra generated by the Mosco topology $\tau_{M_{L^{2}}}$,
(A2) there are functions $\Lambda_{1},\Lambda_{2},\Lambda_{3},\Lambda_{4}:I\times [0,\infty )\to [0,\infty )$ that satisfy the following conditions:
  (i) for every u∈[0,∞), the functions $\Lambda_{1}(\cdot ,u),\Lambda_{2}(\cdot ,u),\Lambda_{3}(\cdot ,u),\Lambda_{4}(\cdot ,u):I\to [0,\infty )$ are integrable,
  (ii) for every t∈I, the functions $\Lambda_{1}(t,\cdot ),\Lambda_{2}(t,\cdot ),\Lambda_{3}(t,\cdot ),\Lambda_{4}(t,\cdot ):[0,\infty )\to [0,\infty )$ are continuous and nondecreasing,
  (iii) $\Lambda_{1}(t,0)=\Lambda_{2}(t,0)=\Lambda_{3}(t,0)=\Lambda_{4}(t,0)=0$ for every t∈I,
  (iv) the following implication with an integral inequality holds:
    if H(0)=0 and $H(t)⩽\sum_{k=1}^{4}C_{k}\int_{0}^{t}\Lambda_{k}(s,H(s))ds$,
    where H:I→[0,∞) and $C_{1},C_{2},C_{3},C_{4}$ are positive constants,
    then H(t)=0 for every t∈I,
  (v) for λ×P-almost all (t,ω) and for every $U,V\in {𝒫}_{c}^{b}(L^{2})$,
    $${𝒟}_{{\mathbb{R}}^{d}}^{2}(A_{k}(t,\omega ,U),A_{k}(t,\omega ,V))⩽\Lambda_{k}(t,{𝒟}_{L^{2}}^{2}(U,V)),\;k=1,2,3,4,$$
(A3) there exist functions $f_{1},f_{2},f_{3},f_{4},h_{1},h_{2},h_{3},h_{4}:I\to [0,\infty )$ that are integrable and satisfy the following condition for λ×P-almost all (t,ω) and for every $U\in {𝒫}_{c}^{b}(L^{2})$:
  $${𝒟}_{{\mathbb{R}}^{d}}^{2}(A_{k}(t,\omega ,U),\{\theta_{{\mathbb{R}}^{d}}\})⩽f_{k}(t)+h_{k}(t){𝒟}_{L^{2}}^{2}(U,\{\theta_{L^{2}}\}),\;k=1,2,3,4,$$
(A4) there exists $\tilde{T}\in (0,T]$ such that the sequence $\{U_{n}{\}}_{n=1}^{\infty }$, defined as
  $$U_{1}(t)=U_{0},\;t\in J=[0,\tilde{T}],$$
  and, for n=2,3,…,
  $$U_{n}(t)=\left[U_{0}+(M)\int_{0}^{t}A_{3}(s,U_{n-1}(s))ds+(M)\int_{0}^{t}A_{4}(s,U_{n-1}(s))dW(s)\right]$$
$$⊖\left[(M)\int_{0}^{t}A_{1}(s,U_{n-1}(s))ds+(M)\int_{0}^{t}A_{2}(s,U_{n-1}(s))d\tilde{W}(s)\right],\;t\in J,$$
  is well defined, particularly that the Hukuhara differences exist.

Conditions (A0) and (A1) ensure the appropriate measurability of mappings and, together with the growth condition (A3), are rather standard requirements imposed on the coefficients of stochastic equations.

Condition (A2), proposed above, is so far the weakest condition imposed on the coefficients of the bilateral multivalued stochastic integral equation to guarantee the existence and uniqueness of its solution. Hence, the examination contained in this paper is the next step after the results obtained with a Lipschitz condition in [36] and with a requirement stronger than the current condition but weaker than the Lipschitz condition, as posed in the paper [37]. In the paper [42] a condition of type (A2) was also considered in the context of single-valued stochastic equations. Referring to it, one can cite a sufficient condition for fulfilling (A2)(iv) along with some examples of the functions $\Lambda_{k}$.

**Remark 1.** *(see [42]). Suppose that the functions $\Lambda_{k}(\cdot ,u):I\to [0,\infty )$ are integrable for every u∈[0,∞), the functions $\Lambda_{k}(t,\cdot ):[0,\infty )\to [0,\infty )$ are continuous and do not decrease for every t∈I, $\Lambda_{k}(t,0)=0$ for every t∈I, u(·) is a solution of the differential equation $\frac{d}{dt}u(t)=\sum_{k=1}^{4}\Lambda_{k}(t,u(t))$, and is satisfied: if there exists $t^{*}\in [0,T)$ such that u(t^*^) = 0, then u(t)=0 for every $t\in [t^{*},T]$. Then, the following implication holds: if a continuous function H:I→[0,∞) satisfies H(0)=0 and*


$$H(t)⩽\sum_{k=1}^{4}C_{k}\int_{0}^{t}\Lambda_{k}(s,H(s))ds\;\text{for every}\;t\in I,$$

*where C_k_>0, then H(t)=0 for every t∈I.*

**Remark 2.** *(see [42]). A sample of functions $\Lambda_{k}$ that meet condition (A2)(iv) can be given as $\Lambda_{k}(t,u)=\alpha (t)\beta (u)$, where α:I→[0,∞) is integrable and β:[0,∞)→[0,∞) does not decrease and is continuous, β(0)=0, and $\int_{0^{+}}\frac{1}{\beta (u)}du=\infty$.*

Observe that if α≡L, where *L* is a positive constant, and β(u)=u, then we obtain the Lipschitz condition from the assumption (A2)(v) discussed in [36]. Moreover, if α≡L, with *L* being a positive constant, and β as described in Remark 2 and concave, we derive a condition utilized in [37]. These observations clearly demonstrate that condition (A2) used in this paper is more general and allows for the development of results concerning bilateral multivalued stochastic integral equations. Apart from the aforementioned example of the function β as β(u)=u, references to more complex functions can be found in the literature [43]. These include, for instance, the functions $\beta_{1},\beta_{2}$ defined as follows:


$$\beta_{1}(u)=\left\{ \begin{array}{c} u\ln\frac{1}{u},\;0⩽u⩽\varepsilon ,\\ \varepsilon \ln\frac{1}{\varepsilon }+\beta_{1}^{\prime }(\varepsilon -)(u-\varepsilon ),\;u>\varepsilon , \end{array}\right.$$

$$\beta_{2}(u)=\left\{ \begin{array}{c} u\ln\frac{1}{u}\ln\ln\frac{1}{u},\;0⩽u⩽\varepsilon ,\\ \varepsilon \ln\frac{1}{\varepsilon }\ln\ln\frac{1}{\varepsilon }+\beta_{2}^{\prime }(\varepsilon -)(u-\varepsilon ),\;u>\varepsilon , \end{array}\right.$$

where ε∈(0,1) is sufficiently small, $\beta_{1}^{\prime }(\varepsilon -),\beta_{2}^{\prime }(\varepsilon -)$ denote left-sided derivatives of $\beta_{1}$ and $\beta_{2}$, respectively, at ε.

The condition (A4), which may seem unfamiliar in studies of right-sided unilateral Eq (2) that mimic the form of single-valued equations, is, however, indispensable and irremovable when studying left-sided unilateral Eq (3) or bilateral Eq (10). This statement may also be justified by the following slightly different formulation of the Eq (10):

$$U(t)=[U_{0}+(M)\int_{0}^{t}A_{3}(s,U(s))\;ds+(M)\int_{0}^{t}A_{4}(s,U(s))\;dW(s)]$$ (11)

$$⊖[(M)\int_{0}^{t}A_{1}(s,U(s))\;ds+(M)\int_{0}^{t}A_{2}(s,U(s))\;d\tilde{W}(s)].$$

It is evident that it involves certain Hukuhara differences, which present some problematic issues, as mentioned in the Preliminaries. The Hukuhara difference may not exist. Therefore, such an assumption (A4) must be made. In the research conducted in this paper, the problem of Hukuhara differences becomes even more significant because the sets to be subtracted come from the hyperspace of subsets of square-integrable random variables, i.e., subsets of an infinite-dimensional space. There is no simple criterion for checking the existence of Hukuhara differences in subsets of such a space. This poses some limitations but also challenges and opens new research directions in the area of equations considered in this paper also for other researchers interested in this topic. To make a step in this direction, in the last research section of this paper, we will show a particular type of bilateral equation and prove the existence of Hukuhara differences, but this will be done for subsets of a finite-dimensional space. Regrettably, verifying the existence of differences for subsets within the abstract space ${\text{L}}^{2}$ remains a highly challenging endeavor and for the time being, condition (A4) must remain.

After an extensive discussion of the two most important conditions (A2) and (A4), the analysis of Eq (10) will now be presented.

We will use the sequence {*U*_*n*_}, described in (A4), to prove the existence and uniqueness of the solution for equations of type (10). However, at the outset we will address the elementary issue of the rationale that the mappings *U*_*n*_ are well defined multivalued mappings.

**Lemma 2.** *Assume that conditions (A0), (A1), (A3), and (A4) hold. Then each mapping $U_{n}:J\to {𝒫}_{c}^{b}(L^{2})$ is well defined and continuous with respect to the metric ${𝒟}_{L^{2}}$.*

**Proof.** The mapping $U_{1}(t)\equiv U_{0}$ is trivially well defined and continuous. Considering this and assuming (A2), we find that for k=1,2,3,4, the composition $A_{k}(\cdot ,\cdot ,U_{1}(\cdot )):I\times \Omega \to {𝒫}_{c}^{b}({\mathbb{R}}^{d})$ forms a nonanticipating multivalued stochastic process. By applying assumption (A3), we have


$${𝒟}_{{\mathbb{R}}^{d}}^{2}(A_{k}(t,\omega ,U_{1}(t)),\{\theta_{{\mathbb{R}}^{d}}\})⩽f_{k}(t)+h_{k}(t){𝒟}_{L^{2}}^{2}(U_{0},\{\theta_{L^{2}}\}).$$

Using assumption (A0), the integrability of *f*_*k*_ and *h*_*k*_, we infer the $L_{𝒩}^{2}(\lambda \;\times \;P)$-integrally boundedness of $A_{k}(\cdot ,\cdot ,U_{1}(\cdot ))$. Therefore, *U*_2_ is well defined, and $U_{2}(t)\in {𝒫}_{c}^{b}(L_{t}^{2})$ for t∈J. Naturally, $U_{2}:J\to {𝒫}_{c}^{b}(L^{2})$ is continuous with respect to ${𝒟}_{L^{2}}$.

Recall that the topology induced by the metric ${𝒟}_{L^{2}}$ is stronger than the Mosco topology $\tau_{M_{L^{2}}}$. Therefore, $U_{2}:J\to {𝒫}_{c}^{b}(L^{2})$ remains continuous when ${𝒫}_{c}^{b}(L^{2})$ is equipped with Mosco topology $\tau_{M_{L^{2}}}$. This observation, combined with assumption (A1), allows us to deduce that the mapping $\left(t,\omega )\mapsto A_{k}(t,\omega ,U_{2}(t)\right)$ is a nonanticipating multivalued stochastic process. For the integrals on the right-hand side of *U*_3_ to be well defined, we must demonstrate that the composition $\left(t,\omega )\mapsto A_{k}(t,\omega ,U_{2}(t)\right)$ is $L_{𝒩}^{2}(\lambda \times P)$-integrally bounded. However, by invoking assumption (A3) again, we can write


$${𝒟}_{{\mathbb{R}}^{d}}^{2}(A_{k}(t,\omega ,U_{2}(t)),\{\theta_{{\mathbb{R}}^{d}}\})⩽f_{k}(t)+h_{k}(t){𝒟}_{L^{2}}^{2}(U_{2}(t),\{\theta_{L^{2}}\}),$$

and thanks to the ${𝒟}_{L^{2}}$-continuity of *U*_2_ and the integrability of *f*_*k*_ and *h*_*k*_, we achieve the desired $L_{𝒩}^{2}(\lambda \;\times \;P)$-integrally boundedness of $A_{k}(\cdot ,\cdot ,U_{2}(\cdot ))$. Due to assumption (A4), the appropriate Hukuhara differences exist, allowing us to assert that *U*_3_ is well defined and continuous. Similarly, the definition of each $U_{4},U_{5},\ldots$ is correct. ◻

All mappings *U*_*n*_ are ${𝒟}_{L^{2}}$-continuous and defined over a closed and bounded interval *J*. Let $q_{n}=\sup_{t\in J}{𝒟}_{L^{2}}(U_{n}(t),\{\theta_{L^{2}}\})$ for n∈ℕ. Naturally, the sequence {*q*_*n*_} is bounded from below. In the following lemma, we establish that the sequence {*q*_*n*_} is also bounded from above.

**Lemma 3.** *Assume that the conditions (A0), (A1), (A3), and (A4) hold. Then there exists a positive constant M such that for every n=1,2,…*


$$\underset{t\in J}{\sup}{𝒟}_{L^{2}}^{2}(U_{n}(t),\{\theta_{L^{2}}\})⩽M.$$

**Proof.** For n∈ℕ and t∈J we have


$$\underset{u\in [0,t]}{\sup}{𝒟}_{L^{2}}^{2}(U_{n}(u),\{\theta_{L^{2}}\})$$

$$=\underset{u\in [0,t]}{\sup}{𝒟}_{L^{2}}^{2}(\left[U_{0}+(M)\int_{0}^{u}A_{3}(s,U_{n-1}(s))ds+(M)\int_{0}^{u}A_{4}(s,U_{n-1}(s))dW(s)\right]$$

$$⊖\left[(M)\int_{0}^{u}A_{1}(s,U_{n-1}(s))ds+(M)\int_{0}^{u}A_{2}(s,U_{n-1}(s))d\tilde{W}(s)\right],\{\theta_{L^{2}}\}).$$

Using property (P1) we get


$$\underset{u\in [0,t]}{\sup}{𝒟}_{L^{2}}^{2}(U_{n}(u),\{\theta_{L^{2}}\})$$

$$=\underset{u\in [0,t]}{\sup}{𝒟}_{L^{2}}^{2}(U_{0}+(M)\int_{0}^{u}A_{3}(s,U_{n-1}(s))ds+(M)\int_{0}^{u}A_{4}(s,U_{n-1}(s))dW(s),$$

$$(M)\int_{0}^{u}A_{1}(s,U_{n-1}(s))ds+(M)\int_{0}^{u}A_{2}(s,U_{n-1}(s))d\tilde{W}(s)).$$

By (P2) and the Cauchy–Buniakowski–Schwarz inequality


$$\;\underset{u\in [0,t]}{\sup}{𝒟}_{L^{2}}^{2}(U_{n}(u),\{\theta_{L^{2}}\})\;\;⩽\;3{𝒟}_{L^{2}}^{2}(U_{0},\{\theta_{L^{2}}\})\;\;+\;3\underset{u\in [0,t]}{\sup}{𝒟}_{L^{2}}^{2}\left((M)\int_{0}^{u}A_{1}(s,U_{n-1}(s))ds,(M)\int_{0}^{u}A_{3}(s,U_{n-1}(s))ds\right)\;\;+\;3\underset{u\in [0,t]}{\sup}{𝒟}_{L^{2}}^{2}\left((M)\int_{0}^{u}A_{2}(s,U_{n-1}(s))d\tilde{W}(s),(M)\int_{0}^{u}A_{4}(s,U_{n-1}(s))dW(s)\right).$$

Invoking properties of multivalued integrals in Lemma 1(i)


$$\;\underset{u\in [0,t]}{\sup}{𝒟}_{L^{2}}^{2}(U_{n}(u),\{\theta_{L^{2}}\})\;\;⩽\;3{𝒟}_{L^{2}}^{2}(U_{0},\{\theta_{L^{2}}\})\;\;+\;3\underset{u\in [0,t]}{\sup}u\int_{[0,u]\times \Omega }{𝒟}_{{\mathbb{R}}^{d}}^{2}(A_{1}(s,\omega ,U_{n-1}(s)),A_{3}(s,\omega ,U_{n-1}(s)))ds\times dP\;\;+\;3\underset{u\in [0,t]}{\sup}\int_{[0,u]\times \Omega }{𝒟}_{{\mathbb{R}}^{d}}^{2}(A_{2}(s,\omega ,U_{n-1}(s)),A_{4}(s,\omega ,U_{n-1}(s)))ds\times dP\;\;=\;3{𝒟}_{L^{2}}^{2}(U_{0},\{\theta_{L^{2}}\})+3t\int_{[0,t]\times \Omega }{𝒟}_{{\mathbb{R}}^{d}}^{2}(A_{1}(s,\omega ,U_{n-1}(s)),A_{3}(s,\omega ,U_{n-1}(s)))ds\times dP\;\;+\;3\int_{[0,t]\times \Omega }{𝒟}_{{\mathbb{R}}^{d}}^{2}(A_{2}(s,\omega ,{\tilde{U}}_{n-1}(s)),A_{4}(s,\omega ,{\tilde{U}}_{n-1}(s)))ds\times dP.$$

By triangle inequality and Cauchy–Buniakowski–Schwarz inequality we get


$$\;\underset{u\in [0,t]}{\sup}{𝒟}_{L^{2}}^{2}(U_{n}(t),\{\theta_{L^{2}}\})\;\;⩽3{𝒟}_{L^{2}}^{2}(U_{0},\{\theta_{L^{2}}\})\;+\;6t\int_{[0,t]\times \Omega }{𝒟}_{{\mathbb{R}}^{d}}^{2}(A_{1}(s,\omega ,U_{n-1}(s)),\{\theta_{{\mathbb{R}}^{d}}\})ds\times dP\;+\;6t\int_{[0,t]\times \Omega }{𝒟}_{{\mathbb{R}}^{d}}^{2}(A_{3}(s,\omega ,U_{n-1}(s)),\{\theta_{{\mathbb{R}}^{d}}\})ds\times dP\;+\;6\int_{[0,t]\times \Omega }{𝒟}_{{\mathbb{R}}^{d}}^{2}(A_{2}(s,\omega ,U_{n-1}(s)),\{\theta_{{\mathbb{R}}^{d}}\})ds\times dP\;+\;6\int_{[0,t]\times \Omega }{𝒟}_{{\mathbb{R}}^{d}}^{2}(A_{4}(s,\omega ,U_{n-1}(s)),\{\theta_{{\mathbb{R}}^{d}}\})ds\times dP.$$

Invoking assumption (A3) one can write


$$\;\underset{u\in [0,t]}{\sup}{𝒟}_{L^{2}}^{2}(U_{n}(u),\{\theta_{L^{2}}\})\;\;⩽3{𝒟}_{L^{2}}^{2}(U_{0},\{\theta_{L^{2}}\})+6t\int_{0}^{t}(f_{1}(s)+f_{3}(s))ds\;+\;6\int_{0}^{t}(f_{2}(s)+f_{4}(s))ds\;+\;6t\int_{0}^{t}(h_{1}(s)+h_{3}(s)){𝒟}_{L^{2}}^{2}(U_{n-1}(s),\{\theta_{L^{2}}\})ds\;+\;6\int_{0}^{t}(h_{2}(s)+h_{4}(s)){𝒟}_{L^{2}}^{2}(U_{n-1}(s),\{\theta_{L^{2}}\})ds.$$

Hence for every l∈ℕ


$$\;\max_{1⩽n⩽l}\underset{u\in [0,t]}{\sup}{𝒟}_{L^{2}}^{2}(U_{n}(u),\{\theta_{L^{2}}\})\;⩽\;3{𝒟}_{L^{2}}^{2}(U_{0},\{\theta_{L^{2}}\})+6t\int_{0}^{t}(f_{1}(s)+f_{3}(s))ds+6\int_{0}^{t}(f_{2}(s)+f_{4}(s))ds\;+\;6t\int_{0}^{t}(h_{1}(s)+h_{3}(s))\max_{1⩽n⩽l}\underset{u\in [0,s]}{\sup}{𝒟}_{L^{2}}^{2}(U_{n-1}(u),\{\theta_{L^{2}}\})ds\;+\;6\int_{0}^{t}(h_{2}(s)+h_{4}(s))\max_{1⩽n⩽l}\underset{u\in [0,s]}{\sup}{𝒟}_{L^{2}}^{2}(U_{n-1}(u),\{\theta_{L^{2}}\})ds.$$

Since $\max_{1⩽n⩽l}\underset{u\in [0,s]}{\sup}{𝒟}_{L^{2}}^{2}(U_{n-1}(u),\{\theta_{L^{2}}\})⩽{𝒟}_{L^{2}}^{2}(U_{0},\{\theta_{L^{2}}\})+\max_{1⩽n⩽l}\underset{u\in [0,s]}{\sup}{𝒟}_{L^{2}}^{2}(U_{n}(u),\{\theta_{L^{2}}\})$, we obtain


$$\;\max_{1⩽n⩽l}\underset{u\in [0,t]}{\sup}{𝒟}_{L^{2}}^{2}(U_{n}(u),\{\theta_{L^{2}}\})\;⩽{𝒟}_{L^{2}}^{2}(U_{0},\{\theta_{L^{2}}\})[3+6t\int_{0}^{t}(h_{1}(s)+h_{3}(s))ds\;+\;6\int_{0}^{t}(h_{2}(s)+h_{4}(s))ds]+6t\int_{0}^{t}(f_{1}(s)+f_{3}(s))ds+6\int_{0}^{t}(f_{2}(s)+f_{4}(s))ds\;+\;6t\int_{0}^{t}(h_{1}(s)+h_{3}(s))\max_{1⩽n⩽l}\underset{u\in [0,s]}{\sup}{𝒟}_{L^{2}}^{2}(U_{n}(u),\{\theta_{L^{2}}\})ds\;+\;6\int_{0}^{t}(h_{2}(s)+h_{4}(s))\max_{1⩽n⩽l}\underset{u\in [0,s]}{\sup}{𝒟}_{L^{2}}^{2}(U_{n}(u),\{\theta_{L^{2}}\})ds.$$

By the Gronwall inequality


$$\;\max_{1⩽n⩽l}\underset{u\in [0,t]}{\sup}{𝒟}_{L^{2}}^{2}(U_{n}(u),\{\theta_{L^{2}}\})\;⩽({𝒟}_{L^{2}}^{2}(U_{0},\{\theta_{L^{2}}\})[3+6\tilde{T}\int_{J}(h_{1}(s)+h_{3}(s))ds\;+\;6\int_{J}(h_{2}(s)+h_{4}(s))ds]+6\tilde{T}\int_{J}(f_{1}(s)+f_{3}(s))ds+6\int_{J}(f_{2}(s)+f_{4}(s))ds)\;\times \exp\{\int_{0}^{t}[6\tilde{T}(h_{1}(s)+h_{3}(s))+6(h_{2}(s)+h_{4}(s))]ds\}.$$

for every l∈ℕ and t∈J. From this it follows that


$$\sup_{u\in J}{𝒟}_{L^{2}}^{2}(U_{n}(u),\{\theta_{L^{2}}\})⩽M,$$

where


$$M=({𝒟}_{L^{2}}^{2}(U_{0},\{\theta_{L^{2}}\})[3+6\tilde{T}\int_{J}(h_{1}(s)+h_{3}(s))ds\;+\;6\int_{J}(h_{2}(s)+h_{4}(s))ds]+6\tilde{T}\int_{J}(f_{1}(s)+f_{3}(s))ds+6\int_{J}(f_{2}(s)+f_{4}(s))ds)$$

$$\times \exp\{\int_{J}[6\tilde{T}(h_{1}(s)+h_{3}(s))+6(h_{2}(s)+h_{4}(s))]ds\}.$$

◻

Let ℭ represent the set of all ${𝒟}_{L^{2}}$-continuous mappings from the interval *J* to the set ${𝒫}_{c}^{b}(L^{2})$, and let 𝔪 be the supremum metric in ℭ, defined as


$$𝔪(U,V)=\sup_{t\in J}{𝒟}_{L^{2}}(U(t),V(t))\;\text{for }U,V\in ℭ.$$

It is well known that the metric space (ℭ,𝔪) is complete. Each mapping *U*_*n*_ described in assumption (A4) is an element of this space. Our next assertion is that {*U*_*n*_} forms a Cauchy sequence in (ℭ,𝔪).

**Lemma 4.** *Assume that the conditions (A0)–(A4) are satisfied. Then, for the sequence {U_n_} defined in (A4), we have*


$$𝔪(U_{n},U_{\ell })⟶0,\;\text{as}\;n,\ell \to \infty .$$

**Proof.** For n,ℓ∈ℕ and t∈J we have


$$\;\underset{u\in [0,t]}{\sup}{𝒟}_{L^{2}}^{2}(U_{n+1}(u),U_{\ell +1}(u))\;=\;\underset{u\in [0,t]}{\sup}{𝒟}_{L^{2}}^{2}([U_{0}+(M)\int_{0}^{u}A_{3}(s,U_{n}(s))ds\;+\;(M)\int_{0}^{u}A_{4}(s,U_{n}(s))dW(s)]\;⊖[(M)\int_{0}^{u}A_{1}(s,U_{n}(s))ds+(M)\int_{0}^{u}A_{2}(s,U_{n}(s))d\tilde{W}(s)],\;[U_{0}+(M)\int_{0}^{u}A_{3}(s,U_{\ell }(s))ds\;+\;(M)\int_{0}^{u}A_{4}(s,U_{\ell }(s))dW(s)]\;⊖[(M)\int_{0}^{u}A_{1}(s,U_{\ell }(s))ds+(M)\int_{0}^{u}A_{2}(s,U_{\ell }(s))d\tilde{W}(s)])\;⩽4t\int_{[0,t]\times \Omega }{𝒟}_{{\mathbb{R}}^{d}}^{2}(A_{3}(s,\omega ,U_{n}(s)),A_{3}(s,\omega ,U_{\ell }(s)))ds\times dP\;+\;4\int_{[0,t]\times \Omega }{𝒟}_{{\mathbb{R}}^{d}}^{2}(A_{4}(s,\omega ,U_{n}(s)),A_{4}(s,\omega ,U_{\ell }(s)))ds\times dP\;+\;4t\int_{[0,t]\times \Omega }{𝒟}_{{\mathbb{R}}^{d}}^{2}(A_{1}(s,\omega ,U_{n}(s)),A_{1}(s,\omega ,U_{\ell }(s)))ds\times dP\;+\;4\int_{[0,t]\times \Omega }{𝒟}_{{\mathbb{R}}^{d}}^{2}(A_{2}(s,\omega ,U_{n}(s)),A_{2}(s,\omega ,U_{\ell }(s)))ds\times dP$$

By assumption (A2) we obtain


$$\;\underset{u\in [0,t]}{\sup}{𝒟}_{L^{2}}^{2}(U_{n+1}(u),U_{\ell +1}(u))\;⩽\;4t\int_{0}^{t}\Lambda_{3}(s,{𝒟}_{L^{2}}^{2}(U_{n}(s),U_{\ell }(s)))ds+4\int_{0}^{t}\Lambda_{4}(s,{𝒟}_{L^{2}}^{2}(U_{n}(s),U_{\ell }(s)))ds\;+\;4t\int_{0}^{t}\Lambda_{1}(s,{𝒟}_{L^{2}}^{2}(U_{n}(s),U_{\ell }(s)))ds+4\int_{0}^{t}\Lambda_{2}(s,{𝒟}_{L^{2}}^{2}(U_{n}(s),U_{\ell }(s)))ds.$$

Since the functions $\Lambda_{k}(s,\cdot )$ are nondecreasing, we get

$$\;\underset{u\in [0,t]}{\sup}{𝒟}_{L^{2}}^{2}(U_{n}(u),U_{\ell }(u))\;⩽\;4t\int_{0}^{t}\Lambda_{3}(s,\underset{u\in [0,s]}{\sup}{𝒟}_{L^{2}}^{2}(U_{n}(u),U_{\ell }(u)))ds\;+\;4\int_{0}^{t}\Lambda_{4}(s,\underset{u\in [0,s]}{\sup}{𝒟}_{L^{2}}^{2}(U_{n}(u),U_{\ell }(u)))ds\;+\;4t\int_{0}^{t}\Lambda_{1}(s,\underset{u\in [0,s]}{\sup}{𝒟}_{L^{2}}^{2}(U_{n}(u),U_{\ell }(u)))ds\;+\;4\int_{0}^{t}\Lambda_{2}(s,\underset{u\in [0,s]}{\sup}{𝒟}_{L^{2}}^{2}(U_{n}(u),U_{\ell }(u)))ds.$$ (12)

Let us denote


$$H(t):={\overset{―}{\lim}}_{n,\ell \to \infty }\underset{u\in [0,t]}{\sup}{𝒟}_{L^{2}}^{2}(U_{n}(u),U_{\ell }(u))\;\text{for}\;\;t\in J.$$

Using inequality (12), the Fatou lemma, and the fact that $\Lambda_{k}(s,\cdot )$ is a continuous function, we arrive at inequality


$$H(t)⩽4\tilde{T}\int_{0}^{t}\Lambda_{3}(s,H(s))ds+4\int_{0}^{t}\Lambda_{4}(s,H(s))ds\;+\;4\tilde{T}\int_{0}^{t}\Lambda_{1}(s,H(s))ds+4\int_{0}^{t}\Lambda_{2}(s,H(s))ds.$$

By assuming (A2) we obtain H(t)=0 for t∈J. From this we conclude that


$$\lim_{n,\ell \to \infty }\underset{u\in [0,t]}{\sup}{𝒟}_{L^{2}}^{2}(U_{n}(u),U_{\ell }(u))=0\;\text{for}\;\;t\in J.$$

Taking $t=\tilde{T}$ we get $\lim_{n,\ell \to \infty }𝔪(U_{n},U_{\ell })=0$. ◻

As introduced in assumption (A4), the sequence {*U*_*n*_} has a limit U∈ℭ, as indicated by the above lemma. This limit will be a solution to Eq (10). This is described in the following statement.

**Theorem 1.** *Assume that conditions (A0)–(A4) hold. The limit U of the sequence {U_n_} is the unique solution to Eq (10).*

**Proof.** As we mentioned and proved in Lemma 4, sequence {*U*_*n*_} is a Cauchy sequence in (C,m) and possesses the limit U∈ℭ, i.e.

$$𝔪(U_{n},U)=\sup_{t\in J}{𝒟}_{L^{2}}(U_{n}(t),U(t))⟶0,\;\text{as}\;n\to \infty .$$ (13)

To justify that *U* is indeed the solution of (10), it is enough to show that

$$\;{𝒟}_{L^{2}}(U(t),\left[U_{0}+(M)\int_{0}^{t}A_{3}(s,U(s))ds+(M)\int_{0}^{t}A_{4}(s,U(s))dW(s)\right]\;⊖\left[(M)\int_{0}^{t}A_{1}(s,U(s))ds+(M)\int_{0}^{t}A_{2}(s,U(s))d\tilde{W}(s)\right])=0.$$ (14)

Due to triangle inequality and Cauchy–Buniakowski–Schwarz inequality we have for t∈J


$$\;{𝒟}_{L^{2}}^{2}(U(t),\left[U_{0}+(M)\int_{0}^{t}A_{3}(s,U(s))ds+(M)\int_{0}^{t}A_{4}(s,U(s))dB(s)\right]\;⊖\left[(M)\int_{0}^{t}A_{1}(s,U(s))ds+(M)\int_{0}^{t}A_{2}(s,U(s))d\tilde{W}(s)\right])\;⩽2{𝒟}_{L^{2}}^{2}\left(U(t),U_{n}(t)\right)+2{𝒟}_{L^{2}}^{2}(U_{n}(t),[U_{0}+(M)\int_{0}^{t}A_{3}(s,U(s))ds\;+\;(M)\int_{0}^{t}A_{4}(s,U(s))dW(s)]⊖[(M)\int_{0}^{t}A_{1}(s,U(s))ds\;+\;(M)\int_{0}^{t}A_{2}(s,U(s))d\tilde{W}(s)]).$$

The first of the components on the right side converges to zero due to (13). In order, we will deal with the second component denoted as *S*_*n*_(*t*) and show that it also converges to zero. Firstly, observe that


$$S_{n}(t)=2{𝒟}_{L^{2}}^{2}([U_{0}+(M)\int_{0}^{t}A_{3}(s,U_{n-1}(s))ds+(M)\int_{0}^{t}A_{4}(s,U_{n-1}(s))dW(s)]\;⊖[(M)\int_{0}^{t}A_{1}(s,U_{n-1}(s))ds+(M)\int_{0}^{t}A_{2}(s,U_{n-1}(s))d\tilde{W}(s)],\;[U_{0}+(M)\int_{0}^{t}A_{3}(s,U(s))ds+(M)\int_{0}^{t}A_{4}(s,U(s))dW(s)]\;⊖[(M)\int_{0}^{t}A_{1}(s,U(s))ds+(M)\int_{0}^{t}A_{2}(s,U(s))d\tilde{W}(s)])\;⩽4{𝒟}_{L^{2}}^{2}(U_{0}+(M)\int_{0}^{t}A_{3}(s,U_{n-1}(s))ds+(M)\int_{0}^{t}A_{4}(s,U_{n-1}(s))dW(s),\;U_{0}+(M)\int_{0}^{t}A_{3}(s,U(s))ds+(M)\int_{0}^{t}A_{4}(s,U(s))dW(s))\mathrm{\backslash nonumber}\;+4{𝒟}_{L^{2}}^{2}((M)\int_{0}^{t}A_{1}(s,U_{n-1}(s))ds+(M)\int_{0}^{t}A_{2}(s,U_{n-1}(s))d\tilde{W}(s)],\;(M)\int_{0}^{t}A_{1}(s,U(s))ds+(M)\int_{0}^{t}A_{2}(s,U(s))d\tilde{W}(s)])\;⩽4t\int_{[0,t]\times \Omega }{𝒟}_{{\mathbb{R}}^{d}}^{2}(A_{3}(s,\omega ,U_{n-1}(s)),A_{3}(s,\omega ,U(s)))ds\times dP\;+\;4\int_{[0,t]\times \Omega }{𝒟}_{{\mathbb{R}}^{d}}^{2}(A_{4}(s,\omega ,U_{n-1}(s)),A_{4}(s,\omega ,U(s)))ds\times dP\;+\;4t\int_{[0,t]\times \Omega }{𝒟}_{{\mathbb{R}}^{d}}^{2}(A_{1}(s,\omega ,U_{n-1}(s)),A_{1}(s,\omega ,U(s)))ds\times dP\;+\;4\int_{[0,t]\times \Omega }{𝒟}_{{\mathbb{R}}^{d}}^{2}(A_{2}(s,\omega ,U_{n-1}(s)),A_{2}(s,\omega ,U(s)))ds\times dP\;⩽4t\int_{0}^{t}\Lambda_{3}(s,{𝒟}_{L^{2}}^{2}(U_{n-1}(s),U(s)))ds+4\int_{0}^{t}\Lambda_{4}(s,{𝒟}_{L^{2}}^{2}(U_{n-1}(s),U(s)))ds\;+\;4t\int_{0}^{t}\Lambda_{1}(s,{𝒟}_{L^{2}}^{2}(U_{n-1}(s),U(s)))ds+4\int_{0}^{t}\Lambda_{2}(s,{𝒟}_{L^{2}}^{2}(U_{n-1}(s),U(s)))ds.$$

The distance ${𝒟}_{L^{2}}^{2}(U_{n-1}(s),U(s))$ is bounded for every n∈ℕ and s∈J by the constant 4*M*, where *M* was established in the proof of Lemma 3. So $\Lambda_{k}(s,{𝒟}_{L^{2}}^{2}(U_{n-1}(s),U(s)))⩽\Lambda_{k}(s,4M)$. Since $\Lambda_{k}(\cdot ,4M)$ is integrable, by Lebesgue Dominated Convergence Theorem we obtain


$$\lim_{n\to \infty }S_{n}(t)⩽4t\int_{0}^{t}\lim_{n\to \infty }\Lambda_{3}(s,{𝒟}_{L^{2}}^{2}(U_{n-1}(s),U(s)))ds\;+\;4\int_{0}^{t}\lim_{n\to \infty }\Lambda_{4}(s,{𝒟}_{L^{2}}^{2}(U_{n-1}(s),U(s)))ds\;+\;4t\int_{0}^{t}\lim_{n\to \infty }\Lambda_{1}(s,{𝒟}_{L^{2}}^{2}(U_{n-1}(s),U(s)))ds\;+\;4\int_{0}^{t}\lim_{n\to \infty }\Lambda_{2}(s,{𝒟}_{L^{2}}^{2}(U_{n-1}(s),U(s)))ds.$$

Recalling the assumption of continuity of $\Lambda_{k}(s,\cdot )$ and the assumption $\Lambda_{k}(s,0)=0$, we get that the right side of the above inequality is equal to zero. Having this it is easy to infer that $\lim_{n\to \infty }S_{n}(t)=0$ for every t∈J. In this way we have finished showing the truthfulness of (14). Thus, the limit *U* of the approximation sequence from (A4) is a solution to the Eq (10).

Suppose this equation has a second solution $V:J\to {𝒫}_{c}^{b}(L^{2})$. By carrying out estimates similar to the previous ones, we will get for t∈J


$$\;{𝒟}_{L^{2}}^{2}(U(t),V(t))\;⩽4t\int_{0}^{t}\Lambda_{3}(s,{𝒟}_{L^{2}}^{2}(U(s),V(s)))ds+4\int_{0}^{t}\Lambda_{4}(s,{𝒟}_{L^{2}}^{2}(U(s),V(s)))ds\;+\;4t\int_{0}^{t}\Lambda_{1}(s,{𝒟}_{L^{2}}^{2}(U(s),V(s)))ds+4\int_{0}^{t}\Lambda_{2}(s,{𝒟}_{L^{2}}^{2}(U(s),V(s)))ds.$$

Recalling the assumption (A2) with condition of integral inequality we get that


$${𝒟}_{L^{2}}^{2}\left(U(t),V(t)\right)=0\text{ for every }t\in J,$$

which ends the proof of uniqueness of the solution *U*. ◻

Using convergence (13) and Lemma 3 it is easy to infer that the solution *U* is bounded. More accurate


$$\underset{t\in J}{\sup}{𝒟}_{L^{2}}(U(t),\{\theta_{L^{2}}\})⩽\sqrt{M},$$

where the constant *M* is like in Lemma 3.

**Corollary 1.** *For the solution U to* Eq (10) *it holds*


$$\underset{t\in J}{\sup}{𝒟}_{L^{2}}(U(t),\{\theta_{L^{2}}\})=\lim_{k\to \infty }\underset{t\in J}{\sup}{𝒟}_{L^{2}}(U_{n_{k}}(t),\{\theta_{L^{2}}\}),$$

*where the n_k_’s correspond to indices of the elements of convergent subsequence of the sequence $\left\{q_{n}\}=\{\underset{t\in J}{\sup}{𝒟}_{L^{2}}(U_{n}(t),\{\theta_{L^{2}}\})\right\}$.*

Next, we will demonstrate the property of continuous dependence of the solution on the equation data. Consider equation (10) as well as similar equations with slightly different data, i.e., equations of the form

$$\;V_{m}(t)+(M)\int_{0}^{t}A_{1,m}(s,V_{m}(s))ds+(M)\int_{0}^{t}A_{2,m}(s,V_{m}(s))d\tilde{W}\mathrm{\backslash nonumber}\;\;=V_{0,m}+(M)\int_{0}^{t}A_{3,m}(s,V_{m}(s))ds+(M)\int_{0}^{t}A_{4,m}(s,V_{m}(s))dW(s)\text{ for }t\in I,$$ (15)

where m∈ℕ.

**Theorem 2.** *Let $U_{0},V_{0,m}\in {𝒫}_{c}^{b}(L_{0}^{2})$ for m∈ℕ and $A_{1},A_{2},A_{3},A_{4}:I\times \Omega \times {𝒫}_{c}^{b}(L^{2})\to {𝒫}_{c}^{b}({\mathbb{R}}^{d})$ satisfy conditions (A0)–(A4). Assume that for every m∈ℕ the mappings $A_{1,m},A_{2,m},A_{3,m},A_{4,m}:I\times \Omega \times {𝒫}_{c}^{b}(L^{2})\to {𝒫}_{c}^{b}({\mathbb{R}}^{d})$ satisfy conditions (A0)–(A4) and in particular for k=1,2,3,4 the mappings A_k,m_’s verify (A2) with the same function ${\tilde{\Lambda }}_{k}$ that is independent of m and possesses property that ${\tilde{\Lambda }}_{k}(t,u)⩽{\tilde{f}}_{k}(t)+{\tilde{h}}_{k}(t)u$ for (t,u)∈I×[0,∞), where ${\tilde{f}}_{k},{\tilde{h}}_{k}:I\to [0,\infty )$ are some integrable functions.*

*If*

$$\lim_{m\to \infty }{𝒟}_{L^{2}}(V_{0,m},U_{0})=0$$ (16)

*and for every $U\in {𝒫}_{c}^{b}(L^{2})$*

$$\lim_{m\to \infty }\sum_{k=1}^{4}\int_{I\times \Omega }{𝒟}_{{\mathbb{R}}^{d}}^{2}(A_{k,m}(t,\omega ,U),A_{k}(t,\omega ,U))ds\times dP=0$$ (17)

*then*


$$\lim_{m\to \infty }\underset{t\in J}{\sup}{𝒟}_{L^{2}}(V_{m}(t),U(t))=0.$$

**Proof.** We will first show that there exists a positive constant *R* such that for every m∈ℕ and every t∈J the property ${𝒟}_{L^{2}}^{2}(V_{m}(t),U(t))⩽R$ holds true. To this end, let us note that for fixed m∈ℕ and t∈J


$$\;{𝒟}_{L^{2}}^{2}(V_{m}(t),U(t))\;=\;{𝒟}_{L^{2}}^{2}(\left[V_{0,m}+(M)\int_{0}^{t}A_{3,m}(s,V_{m}(s))ds+(M)\int_{0}^{t}A_{4,m}(s,V_{m}(s))dW(s)\right]\;⊖\;\left[(M)\int_{0}^{t}A_{1,m}(s,V_{m}(s))ds+(M)\int_{0}^{t}A_{2,m}(s,V_{m}(s))d\tilde{W}(s)\right],\;\left[U_{0}+(M)\int_{0}^{t}A_{3}(s,U(s))ds+(M)\int_{0}^{t}A_{4}(s,U(s))dW(s)\right]\;⊖\;\left[(M)\int_{0}^{t}A_{1}(s,U(s))ds+(M)\int_{0}^{t}A_{2}(s,U(s))d\tilde{W}(s)\right])\;⩽2{𝒟}_{L^{2}}^{2}(V_{0,m}+(M)\int_{0}^{t}A_{3,m}(s,V_{m}(s))ds+(M)\int_{0}^{t}A_{4,m}(s,V_{m}(s))dW(s),\;U_{0}+(M)\int_{0}^{t}A_{3}(s,U(s))ds+(M)\int_{0}^{t}A_{4}(s,U(s))dW(s))\;+2{𝒟}_{L^{2}}^{2}((M)\int_{0}^{t}A_{1,m}(s,V_{m}(s))ds+(M)\int_{0}^{t}A_{2,m}(s,V_{m}(s))d\tilde{W}(s),\;(M)\int_{0}^{t}A_{1}(s,U(s))ds+(M)\int_{0}^{t}A_{2}(s,U(s))d\tilde{W}(s))\;⩽6{𝒟}_{L^{2}}^{2}(V_{0,m},U_{0})+6t\int_{[0,t]\times \Omega }{𝒟}_{{\mathbb{R}}^{d}}^{2}(A_{3,m}(s,\omega ,V_{m}(s)),A_{3}(s,\omega ,U(s)))ds\times dP\;+\;6\int_{[0,t]\times \Omega }{𝒟}_{{\mathbb{R}}^{d}}^{2}(A_{4,m}(s,\omega ,V_{m}(s)),A_{4}(s,\omega ,U(s)))ds\times dP\;+\;4t\int_{[0,t]\times \Omega }{𝒟}_{{\mathbb{R}}^{d}}^{2}(A_{1,m}(s,\omega ,V_{m}(s)),A_{1}(s,\omega ,U(s)))ds\times dP\;+\;4\int_{[0,t]\times \Omega }{𝒟}_{{\mathbb{R}}^{d}}^{2}(A_{2,m}(s,\omega ,V_{m}(s)),A_{2}(s,\omega ,U(s)))ds\times dP\;⩽6{𝒟}_{L^{2}}^{2}(V_{0,m},U_{0})+12t\int_{[0,t]\times \Omega }{𝒟}_{{\mathbb{R}}^{d}}^{2}(A_{3,m}(s,\omega ,V_{m}(s)),A_{3,m}(s,\omega ,U(s)))ds\times dP\;+\;12t\int_{[0,t]\times \Omega }{𝒟}_{{\mathbb{R}}^{d}}^{2}(A_{3,m}(s,\omega ,U(s)),A_{3}(s,\omega ,U(s)))ds\times dP\;+\;12\int_{[0,t]\times \Omega }{𝒟}_{{\mathbb{R}}^{d}}^{2}(A_{4,m}(s,\omega ,V_{m}(s)),A_{4,m}(s,\omega ,U(s)))ds\times dP\;+\;12\int_{[0,t]\times \Omega }{𝒟}_{{\mathbb{R}}^{d}}^{2}(A_{4,m}(s,\omega ,U(s)),A_{4}(s,\omega ,U(s)))ds\times dP\;+\;8t\int_{[0,t]\times \Omega }{𝒟}_{{\mathbb{R}}^{d}}^{2}(A_{1,m}(s,\omega ,V_{m}(s)),A_{1,m}(s,\omega ,U(s)))ds\times dP\;+\;8t\int_{[0,t]\times \Omega }{𝒟}_{{\mathbb{R}}^{d}}^{2}(A_{1,m}(s,\omega ,U(s)),A_{1}(s,\omega ,U(s)))ds\times dP\;+\;8\int_{[0,t]\times \Omega }{𝒟}_{{\mathbb{R}}^{d}}^{2}(A_{2,m}(s,\omega ,V_{m}(s)),A_{2,m}(s,\omega ,U(s)))ds\times dP\;+\;8\int_{[0,t]\times \Omega }{𝒟}_{{\mathbb{R}}^{d}}^{2}(A_{2,m}(s,\omega ,U(s)),A_{2}(s,\omega ,U(s)))ds\times dP.$$

Let *C*_1_ and *C*_2_ be the positive constants such that


$$\sup_{m\in \mathbb{N}}\{6{𝒟}_{L^{2}}^{2}(V_{0,m},U_{0})\}⩽C_{1}$$

and


$$\sup_{m\in \mathbb{N}}\{\sum_{k=1}^{4}\int_{I\times \Omega }{𝒟}_{{\mathbb{R}}^{d}}^{2}(A_{k,m}(t,\omega ,U),A_{k}(t,\omega ,U))ds\times dP\}⩽C_{2}.$$

Then


$$\;{𝒟}_{L^{2}}^{2}(V_{m}(t),U(t))\;⩽C_{1}+20(\tilde{T}+1)C_{2}+12\tilde{T}\int_{0}^{t}{\tilde{\Lambda }}_{3}(s,{𝒟}_{L^{2}}^{2}(V_{m}(s),U(s)))ds\;+\;12\int_{0}^{t}{\tilde{\Lambda }}_{4}(s,{𝒟}_{L^{2}}^{2}(V_{m}(s),U(s)))ds\;+\;8\tilde{T}\int_{0}^{t}{\tilde{\Lambda }}_{1}(s,{𝒟}_{L^{2}}^{2}(V_{m}(s),U(s)))ds+8\int_{0}^{t}{\tilde{\Lambda }}_{2}(s,{𝒟}_{L^{2}}^{2}(V_{m}(s),U(s)))ds.$$

Using assumption ${\tilde{\Lambda }}_{k}(s,u)⩽{\tilde{f}}_{k}(s)+{\tilde{h}}_{k}(s)u$ we get


$$\;{𝒟}_{L^{2}}^{2}(V_{m}(t),U(t))\;⩽C_{1}+20(\tilde{T}+1)C_{2}+\int_{J}(12\tilde{T}{\tilde{f}}_{3}(s)+12{\tilde{f}}_{4}(s)+8\tilde{T}{\tilde{f}}_{1}(s)+8{\tilde{f}}_{2}(s))ds\;+\;\int_{0}^{t}(12\tilde{T}{\tilde{h}}_{3}(s)+12{\tilde{h}}_{4}(s)+8\tilde{T}{\tilde{h}}_{1}(s)+8{\tilde{h}}_{2}(s)){𝒟}_{L^{2}}^{2}(V_{m}(s),U(s))ds.$$

Therefore, going through the Gronwall inequality, we conclude that for every m∈ℕ and every t∈J


$${𝒟}_{L^{2}}^{2}(V_{m}(t),U(t))⩽R,$$

where


$$R=[C_{1}+20(\tilde{T}+1)C_{2}+\int_{J}(12\tilde{T}{\tilde{f}}_{3}(s)+12{\tilde{f}}_{4}(s)+8\tilde{T}{\tilde{f}}_{1}(s)+8{\tilde{f}}_{2}(s))ds]\;\times \exp\{\int_{J}(12\tilde{T}{\tilde{h}}_{3}(s)+12{\tilde{h}}_{4}(s)+8\tilde{T}{\tilde{h}}_{1}(s)+8{\tilde{h}}_{2}(s))ds\}.$$

Hence

$${\tilde{\Lambda }}_{k}(s,{𝒟}_{L^{2}}^{2}(V_{m}(s),U(s)))⩽{\tilde{\Lambda }}_{k}(s,R)\;\text{for each }\;\;s\in J.$$ (18)

By performing calculations similar to those in the earlier part of the proof, we will get that for every t∈J


$$\;\underset{u\in [0,t]}{\sup}{𝒟}_{L^{2}}^{2}(V_{m}(u),U(u))\;⩽6{𝒟}_{L^{2}}^{2}(V_{0,m},U_{0})+12\tilde{T}\int_{0}^{t}{\tilde{\Lambda }}_{3}(s,\underset{u\in [0,s]}{\sup}{𝒟}_{L^{2}}^{2}(V_{m}(u),U(u)))ds\;+\;12\tilde{T}\int_{J\times \Omega }{𝒟}_{{\mathbb{R}}^{d}}^{2}(A_{3,m}(s,\omega ,U(s)),A_{3}(s,\omega ,U(s)))ds\times dP\;+\;12\int_{0}^{t}{\tilde{\Lambda }}_{4}(s,\underset{u\in [0,s]}{\sup}{𝒟}_{L^{2}}^{2}(V_{m}(u),U(u)))ds\;+\;12\int_{J\times \Omega }{𝒟}_{{\mathbb{R}}^{d}}^{2}(A_{4,m}(s,\omega ,U(s)),A_{4}(s,\omega ,U(s)))ds\times dP\;+\;8\tilde{T}\int_{0}^{t}{\tilde{\Lambda }}_{1}(s,\underset{u\in [0,s]}{\sup}{𝒟}_{L^{2}}^{2}(V_{m}(u),U(u)))ds\;+\;8\tilde{T}\int_{J\times \Omega }{𝒟}_{{\mathbb{R}}^{d}}^{2}(A_{1,m}(s,\omega ,U(s)),A_{1}(s,\omega ,U(s)))ds\times dP\;+\;8\int_{0}^{t}{\tilde{\Lambda }}_{2}(s,\underset{u\in [0,s]}{\sup}{𝒟}_{L^{2}}^{2}(V_{m}(u),U(u)))ds\;+\;8\int_{J\times \Omega }{𝒟}_{{\mathbb{R}}^{d}}^{2}(A_{2,m}(s,\omega ,U(s)),A_{2}(s,\omega ,U(s)))ds\times dP.$$

Due to convergences (16) and (17), property (18) and the Lebesgue Dominated Convergence Theorem we infer that


$$\;\lim_{m\to \infty }\underset{u\in [0,t]}{\sup}{𝒟}_{L^{2}}^{2}(V_{m}(u),U(u))\;⩽12\tilde{T}\int_{0}^{t}{\tilde{\Lambda }}_{3}(s,\lim_{m\to \infty }\underset{u\in [0,s]}{\sup}{𝒟}_{L^{2}}^{2}(V_{m}(u),U(u)))ds\;+\;12\int_{0}^{t}{\tilde{\Lambda }}_{4}(s,\lim_{m\to \infty }\underset{u\in [0,s]}{\sup}{𝒟}_{L^{2}}^{2}(V_{m}(u),U(u)))ds\;+\;8\tilde{T}\int_{0}^{t}{\tilde{\Lambda }}_{1}(s,\lim_{m\to \infty }\underset{u\in [0,s]}{\sup}{𝒟}_{L^{2}}^{2}(V_{m}(u),U(u)))ds\;+\;8\int_{0}^{t}{\tilde{\Lambda }}_{2}(s,\lim_{m\to \infty }\underset{u\in [0,s]}{\sup}{𝒟}_{L^{2}}^{2}(V_{m}(u),U(u)))ds.$$

Due to assumption (A2) of the integral inequality


$$\lim_{m\to \infty }\underset{u\in [0,t]}{\sup}{𝒟}_{L^{2}}^{2}(V_{m}(u),U(u))=0\;\text{for every}\;\;t\in J$$

which completes the proof. ◻

Theorem 1 about the existence and uniqueness of the solution of Eq (10) is crucial to build the theory of equations considered in the paper, and Theorem 2 gives the property that small changes in the data in the equation give small changes in the solution, which makes the obtained solutions stable. These are the two key theorems that allow us to be sure that when the assumptions are met, we obtain well-posed problems described in terms of bilateral multivalued stochastic integral equations.

## Consequences for deterministic bilateral set-valued integral equations

Bilateral set-valued stochastic integral Eq (10) can be considered as generalization of deterministic bilateral set-valued integral equation of the form

$$U(t)+(M)\int_{0}^{t}A_{1}(s,U(s))\;ds=U_{0}+(M)\int_{0}^{t}A_{2}(s,U(s))\;ds\;\text{for }t\in I,$$ (19)

where $A_{1},A_{2}:I\times {𝒫}_{c}^{b}(\mathbb{R})\to {𝒫}_{c}^{b}(\mathbb{R})$, $U_{0}\in {𝒫}_{c}^{b}(\mathbb{R})$, and the integrals are the set-valued Aumann integrals. This becomes even more apparent when we mention that ℝ can be embedded in the space $L^{2}(\Omega ,𝒜,P;\mathbb{R})$.

If $A_{1}\equiv \{0\}$, then (19) represents an integral form of a set-valued differential equation that was studied in [27]. Therefore, Eq (19) is also interesting in their own right, and this section of the paper will be dedicated to Eq (19).

The analysis of Eq (10) proposed in the previous sections and the results obtained can be applied to obtain analogous results regarding the solutions of Eq (19). These results will be formulated here, and furthermore, it will be shown that the assumption of type (A4) is satisfied in this case. Hence, in the study of the existence of a solution to Eq (19) and the determination of its fundamental properties, we consider that the functions *A*_1_ and *A*_2_, defined as $A_{1},A_{2}:I\times {𝒫}_{c}^{b}(\mathbb{R})\to {𝒫}_{c}^{b}(\mathbb{R})$, satisfy

(H1) for every $U\in {𝒫}_{c}^{b}(\mathbb{R})$ the set-valued mappings $A_{1}(\cdot ,U),A_{2}(\cdot ,U):I\to {𝒫}_{c}^{b}(\mathbb{R})$ are $\beta_{I}$-measurable,
(H2) there are functions $\Lambda_{1},\Lambda_{2}:I\times [0,\infty )\to [0,\infty )$ that satisfy the following conditions:
  (i) for every u∈[0,∞), the functions $\Lambda_{1}(\cdot ,u),\Lambda_{2}(\cdot ,u):I\to [0,\infty )$ are integrable,
  (ii) for every t∈I, the functions $\Lambda_{1}(t,\cdot ),\Lambda_{2}(t,\cdot ):[0,\infty )\to [0,\infty )$ are continuous and nondecreasing,
  (iii) $\Lambda_{1}(t,0)=\Lambda_{2}(t,0)=0$ for every t∈I,
  (iv) the following implication with an integral inequality holds:
    if H(0)=0 and $H(t)⩽C_{1}\int_{0}^{t}\Lambda_{1}(s,H(s))ds+C_{2}\int_{0}^{t}\Lambda_{2}(s,H(s))ds$,
    where H:I→[0,∞) and $C_{1},C_{2}$ are positive constants,
    then H(t)=0 for every t∈I,
  (v) for λ-almost all *t* and for every $U,V\in {𝒫}_{c}^{b}(\mathbb{R})$,
    $${𝒟}_{\mathbb{R}}(A_{1}(t,U),A_{1}(t,V))⩽\Lambda_{1}(t,{𝒟}_{\mathbb{R}}(U,V)),$$
    $${𝒟}_{\mathbb{R}}(A_{2}(t,U),A_{2}(t,V))⩽\Lambda_{2}(t,{𝒟}_{\mathbb{R}}(U,V)),$$
(H3) there exist functions $f_{1},f_{2},h_{1},h_{2}:I\to [0,\infty )$ that are integrable and satisfy the following condition for λ-almost all *t* and for every $U\in {𝒫}_{c}^{b}(\mathbb{R})$:
  $${𝒟}_{\mathbb{R}}(A_{1}(t,U),\{0\})⩽f_{1}(t)+h_{1}(t){𝒟}_{\mathbb{R}}(U,\{0\}),$$
$${𝒟}_{\mathbb{R}}(A_{2}(t,U),\{0\})⩽f_{2}(t)+h_{2}(t){𝒟}_{\mathbb{R}}(U,\{0\}),$$

To demonstrate the existence of a unique solution for Eq (19), one can use a sequence of approximations {*U*_*n*_} described by $U_{0}(t)=U_{0}$ and


$$U_{n}(t)=[U_{0}+(M)\int_{0}^{t}A_{2}(s,U_{n-1}(s))ds]⊖(M)\int_{0}^{t}A_{1}(s,U_{n-1}(s))ds.$$

Among the assumptions listed above, none addresses the existence of Hukuhara differences, a known troublesome issue. Recall that in the second section of this paper, we could not demonstrate the existence of Hukuhara differences because the space was infinite-dimensional, lacking a convenient criterion for verifying the existence of Hukuhara differences. However, when considering the specific finite-dimensional space, namely ℝ, and Eq (19), we can provide an easy-to-check sufficient condition for the existence of Hukuhara differences for elements in ${𝒫}_{c}^{b}(\mathbb{R})$. This condition is as follows:


$$\text{if }\mathrm{diam}\;U⩾\mathrm{diam}V\text{ then }U⊖V\text{ exists,}\;U,V\in {𝒫}_{c}^{b}(\mathbb{R}).$$

We will use this criterion to show that the sequence {*U*_*n*_} is well defined on an interval J⊂I=[0,T], and it is not necessary to formulate an assumption of type (A4).

First, we will show that the Hukuhara differences exist in the definition of *U*_1_. Let us denote $M_{0}:={𝒟}_{\mathbb{R}}(U_{0},\{0\})$ and $\tilde{f_{1}}:=\underset{t\in I}{\sup}f_{1}(t)$, $\tilde{f_{2}}:=\underset{t\in I}{\sup}f_{2}(t)$, $\tilde{h_{1}}:=\underset{t\in I}{\sup}h_{1}(t)$, $\tilde{h_{2}}:=\underset{t\in I}{\sup}h_{2}(t)$.

Observe that for $t\in [0,T_{1}]$, where $T_{1}=\min\{\frac{\mathrm{diam}\;U_{0}}{2(\tilde{f_{1}}+\tilde{h_{1}}M_{0})},T\}$ we have


$$\mathrm{diam}\left((M)\int_{0}^{t}A_{1}(s,U_{0})ds\right)⩽2\int_{0}^{t}{𝒟}_{\mathbb{R}}(A_{1}(s,U_{0}),\{0\})ds\;⩽2\int_{0}^{t}[f_{1}(s)+h_{1}(s){𝒟}_{\mathbb{R}}(U_{0},\{0\})]ds\;⩽2(\tilde{f_{1}}+\tilde{h_{1}}M_{0})t\;⩽\mathrm{diam}\;U_{0}\;⩽\mathrm{diam}\left(U_{0}+(M)\int_{0}^{t}A_{2}(s,U_{0})ds\right).$$

Therefore, the Hukuhara difference $\left[U_{0}+(M)\int_{0}^{t}A_{2}(s,U_{0})\;ds\right]⊖(M)\int_{0}^{t}A_{1}(s,U_{0})\;ds$ exists for every $t\in [0,T_{1}]$, which implies that *U*_1_(*t*) is well defined for $t\in [0,T_{1}]$.

In the next step, our goal is to demonstrate the existence of the Hukuhara differences within the definition of *U*_2_. To achieve this, we will initially establish that there is a positive constant *M*_1_ such that $\underset{t\in [0,T_{1}]}{\sup}{𝒟}_{\mathbb{R}}(U_{1}(t),\{0\})⩽M_{1}$. Let us note that


$${𝒟}_{\mathbb{R}}(U_{1}(t),\{0\})={𝒟}_{\mathbb{R}}(U_{0}+\int_{0}^{t}A_{2}(s,U_{0})ds,\int_{0}^{t}A_{1}(s,U_{0})ds)\;⩽M_{0}+{𝒟}_{\mathbb{R}}(\int_{0}^{t}A_{2}(s,U_{0})ds,\int_{0}^{t}A_{1}(s,U_{0})ds).$$

Using Theorem 1.7.3 of [27] we have further


$${𝒟}_{\mathbb{R}}(U_{1}(t),\{0\})⩽M_{0}+\int_{0}^{t}{𝒟}_{\mathbb{R}}(A_{2}(s,U_{0}),A_{1}(s,U_{0}))ds\;⩽M_{0}+\int_{0}^{t}{𝒟}_{\mathbb{R}}(A_{1}(s,U_{0}),\{0\})ds\;+\;\int_{0}^{t}{𝒟}_{\mathbb{R}}(A_{2}(s,U_{0}),\{0\})ds.$$

By assumption (H3)


$${𝒟}_{\mathbb{R}}(U_{1}(t),\{0\})⩽M_{0}+\int_{0}^{t}[f_{1}(s)+h_{1}(s)M_{0}]ds+\int_{0}^{t}[f_{2}(s)+h_{2}(s)M_{0}]ds$$

$$⩽M_{0}+\int_{0}^{t}[\tilde{f_{1}}+\tilde{f_{2}}+(\tilde{h_{1}}+\tilde{h_{2}})M_{0}]ds$$

$$⩽M_{0}+[\tilde{f_{1}}+\tilde{f_{2}}+(\tilde{h_{1}}+\tilde{h_{2}})M_{0}]t.$$ (20)

Hence $\underset{t\in [0,T_{1}]}{\sup}{𝒟}_{\mathbb{R}}(U_{1}(t),\{0\})⩽M_{1}$, where


$$M_{1}=M_{0}+[\tilde{f_{1}}+\tilde{f_{2}}+(\tilde{h_{1}}+\tilde{h_{2}})M_{0}]T.$$

Let us further observe that for $t\in [0,T_{2}]$, where $T_{2}=\min\left\{\frac{\mathrm{diam}\;U_{0}}{2(\tilde{f_{1}}+\tilde{h_{1}}M_{1})},T\right\}$, we have


$$\mathrm{diam}\left((M)\int_{0}^{t}A_{1}(s,U_{1}(s))ds\right)⩽2\int_{0}^{t}{𝒟}_{\mathbb{R}}(A_{1}(s,U_{1}(s)),\{0\})ds$$

$$⩽2\int_{0}^{t}[f_{1}(s)+h_{1}(s){𝒟}_{\mathbb{R}}(U_{1}(s),\{0\})]ds$$

$$⩽2(\tilde{f_{1}}+\tilde{h_{1}}M_{1})t$$

$$⩽\mathrm{diam}\;U_{0}$$

$$⩽\mathrm{diam}\left(U_{0}+(M)\int_{0}^{t}A_{2}(s,U_{1}(s))ds\right).$$

Hence $U_{2}(t)=[U_{0}+(M)\int_{0}^{t}A_{2}(s,U_{1}(s))ds]⊖(M)\int_{0}^{t}A_{1}(s,U_{1}(s))ds$ is well defined for $t\in [0,T_{2}]$.

In the upcoming stage, we will validate the existence of Hukuhara differences in the definition of *U*_3_. For this purpose, we will initially prove the existence of a positive constant *M*_2_ such that $\underset{t\in [0,T_{2}]}{\sup}{𝒟}_{\mathbb{R}}(U_{2}(t),\{0\})⩽M_{2}$. Let us observe that


$${𝒟}_{\mathbb{R}}(U_{2}(t),\{0\})={𝒟}_{\mathbb{R}}(U_{0}+\int_{0}^{t}A_{2}(s,U_{1}(s))ds,\int_{0}^{t}A_{1}(s,U_{1}(s))ds)\;⩽M_{0}+{𝒟}_{\mathbb{R}}(\int_{0}^{t}A_{2}(s,U_{1}(s))ds,\int_{0}^{t}A_{1}(s,U_{1}(s))ds)\;⩽M_{0}+\int_{0}^{t}{𝒟}_{\mathbb{R}}(A_{2}(s,U_{1}(s)),A_{1}(s,U_{1}(s)))ds\;⩽M_{0}+\int_{0}^{t}{𝒟}_{\mathbb{R}}(A_{1}(s,U_{1}(s)),\{0\})ds\;\;+\;\int_{0}^{t}{𝒟}_{\mathbb{R}}(A_{2}(s,U_{1}(s)),\{0\})ds\;⩽M_{0}+\int_{0}^{t}[f_{1}(s)+h_{1}(s){𝒟}_{\mathbb{R}}(U_{1}(s),\{0\})]ds\;\;+\;\int_{0}^{t}[f_{2}(s)+h_{2}(s){𝒟}_{\mathbb{R}}(U_{1}(s),\{0\})]ds\;⩽M_{0}+\int_{0}^{t}[\tilde{f_{1}}+\tilde{f_{2}}+(\tilde{h_{1}}+\tilde{h_{2}}){𝒟}_{\mathbb{R}}(U_{1}(s),\{0\})]ds\;⩽M_{0}+\int_{0}^{t}[\tilde{f_{1}}+\tilde{f_{2}}\;\;+\;(\tilde{h_{1}}+\tilde{h_{2}})[M_{0}+[\tilde{f_{1}}+\tilde{f_{2}}+(\tilde{h_{1}}+\tilde{h_{2}})M_{0}]s]]ds\;⩽M_{0}+[\tilde{f_{1}}+\tilde{f_{2}}+(\tilde{h_{1}}+\tilde{h_{2}})M_{0}]t\;\;+\;(\tilde{h_{1}}+\tilde{h_{2}})[\tilde{f_{1}}+\tilde{f_{2}}+(\tilde{h_{1}}+\tilde{h_{2}})M_{0}]\frac{t^{2}}{2}.$$

Hence $\underset{t\in [0,T_{2}]}{\sup}{𝒟}_{\mathbb{R}}(U_{2}(t),\{0\})⩽M_{2}$, where


$$M_{2}=M_{0}+[\tilde{f_{1}}+\tilde{f_{2}}+(\tilde{h_{1}}+\tilde{h_{2}})M_{0}]T\;\;+\;(\tilde{h_{1}}+\tilde{h_{2}})[\tilde{f_{1}}+\tilde{f_{2}}+(\tilde{h_{1}}+\tilde{h_{2}})M_{0}]\frac{T^{2}}{2}.$$

Let us further observe that for $t\in [0,T_{3}]$, where $T_{3}=\min\left\{\frac{\mathrm{diam}\;U_{0}}{2(\tilde{f_{1}}+\tilde{h_{1}}M_{2})},T\right\}$, we have


$$\mathrm{diam}\left((M)\int_{0}^{t}A_{1}(s,U_{2}(s))ds\right)⩽2\int_{0}^{t}{𝒟}_{\mathbb{R}}(A_{1}(s,U_{2}(s)),\{0\})ds$$

$$⩽2\int_{0}^{t}[f_{1}(s)+h_{1}(s){𝒟}_{\mathbb{R}}(U_{2}(s),\{0\})]ds$$

$$⩽2(\tilde{f_{1}}+\tilde{h_{1}}M_{2})t$$

$$⩽\mathrm{diam}\;U_{0}$$

$$⩽\mathrm{diam}\left(U_{0}+(M)\int_{0}^{t}A_{2}(s,U_{2}(s))ds\right).$$

Therefore, *U*_3_(*t*) is well defined for $t\in [0,T_{3}]$.

By repeating these steps recursively, we obtain that *U*_*n*_(*t*) is well defined on the interval *[*0,*T*_*n*_*]*, where $T_{n}=\min\left\{\frac{\mathrm{diam}\;U_{0}}{2(\tilde{f_{1}}+\tilde{h_{1}}M_{n-1})},T\right\}$ with the constant *M*_*n*−1_ defined by


$$M_{n-1}=M_{0}+[\tilde{f_{1}}+\tilde{f_{2}}+(\tilde{h_{1}}+\tilde{h_{2}})M_{0}]\;\;\times [T+(\tilde{h_{1}}+\tilde{h_{2}})\frac{T^{2}}{2}+\ldots +(\tilde{h_{1}}+\tilde{h_{2}}{)}^{n-2}\frac{T^{n-1}}{(n-1)!}].$$

The sequence {*M*_*n*_} is increasing and bounded from above, for example, by the number


$$M=M_{0}+[\tilde{f_{1}}+\tilde{f_{2}}+(\tilde{h_{1}}+\tilde{h_{2}})M_{0}]\frac{\exp\{(\tilde{h_{1}}+\tilde{h_{2}})T\}}{\tilde{h_{1}}+\tilde{h_{2}}}.$$

Hence, we conclude that each *U*_*n*_ is well defined at least in the interval $J=[0,\tilde{T}]$, where $\tilde{T}=\min\left\{\frac{\mathrm{diam}\;U_{0}}{2(\tilde{f_{1}}+\tilde{h_{1}}M)},T\right\}$. Therefore, in the examination of this specific Eq (19), the assumption of type (A4) is not required. As a consequence of the above analysis, we obtain the following assertion.

**Corollary 2.** *Suppose conditions (H1),(H3) are satisfied. Then, there exists a positive constant M for which, for any n=1,2,…*


$$\underset{t\in J}{\sup}{𝒟}_{\mathbb{R}}(U_{n}(t),\{0\})⩽M.$$

At this point, we can present several results concerning solutions to Eq (19). The proofs are analogous to those for the solutions of Eq (10), so they will not be repeated here. We start with the theorem on the existence and uniqueness.

**Theorem 3.** *Consider $U_{0}\in {𝒫}_{c}^{b}(\mathbb{R})$, with A_1_ and A_2_ defined as $A_{1},A_{2}:I\times {𝒫}_{c}^{b}(\mathbb{R})\to {𝒫}_{c}^{b}(\mathbb{R})$ satisfying conditions (H1) through (H3). Consequently, Eq (19) possesses a unique (potentially local) solution U.*

Of course, the solution *U* from the preceding theorem is the uniform limit of the sequence {*U*_*n*_}, thus


$$\underset{t\in J}{\sup}{𝒟}_{\mathbb{R}}(U(t),\{0\})⩽M,$$

where *M* is the constant of Corollary 2.

Following this, we highlight the characteristic of the solution’s continuous dependence on the equation data. Take into account Eq (19) along with analogous equations that have marginally varied data, i.e., equations of the form


$$V_{m}(t)+(M)\int_{0}^{t}A_{1,m}(s,V_{m}(s))ds=V_{0,m}+(M)\int_{0}^{t}A_{2,m}(s,V_{m}(s))ds\;\;\text{ for }t\in I,$$

where m∈ℕ.

**Theorem 4.** *Let $U_{0},V_{0,m}\in {𝒫}_{c}^{b}(\mathbb{R})$ for m∈ℕ and let the mappings $A_{1},A_{2}:I\;\times \;{𝒫}_{c}^{b}(\mathbb{R})\to {𝒫}_{c}^{b}(\mathbb{R})$ satisfy conditions (H1)-(H3). Suppose that for every m∈ℕ the mappings $A_{1,m},A_{2,m}:I\times {𝒫}_{c}^{b}(\mathbb{R})\to {𝒫}_{c}^{b}(\mathbb{R})$ also satisfy conditions (H1)–(H3). Moreover, assume that $A_{1,m},A_{2,m}$ satisfy (H2) with the same functions ${\tilde{\Lambda }}_{1},{\tilde{\Lambda }}_{2}$, respectively, which are independent of m and possess the property ${\tilde{\Lambda }}_{k}(t,u)⩽{\tilde{f}}_{k}(t)+{\tilde{h}}_{k}(t)u$ for (t,u)∈I×[0,∞), where ${\tilde{f}}_{k},{\tilde{h}}_{k}:I\to [0,\infty )$ are some integrable functions, k=1,2.*

*If*


$$\lim_{m\to \infty }{𝒟}_{\mathbb{R}}(V_{0,m},U_{0})=0$$

*and for every $U\in {𝒫}_{c}^{b}(\mathbb{R})$*


$$\lim_{m\to \infty }(\int_{I}{𝒟}_{\mathbb{R}}(A_{1,m}(t,U),A_{1}(t,U))ds+\int_{I}{𝒟}_{\mathbb{R}}(A_{2,m}(t,U),A_{2}(t,U))ds)=0$$

*then*


$$\lim_{m\to \infty }\underset{t\in J}{\sup}{𝒟}_{\mathbb{R}}(V_{m}(t),U(t))=0.$$

In this way, we have concluded the identification of the fundamental properties of solutions to deterministic bilateral set-valued integral equations.

## Conclusion

In this paper, we consider a certain hypothetical and imaginative example of stochastic modeling of population growth. We justify how one can transition from such a single-valued equation to a multivalued one. We propose a bilateral multivalued stochastic integral equation that addresses two types of uncertainty in such modeling, namely stochasticity and imprecision. The fundamental question in analyzing such equations is whether they have solutions. We present a collection of conditions that, when satisfied, guarantee the existence of a unique solution. This collection of conditions is the least demanding among those present in the literature for these types of equations. Additionally, we demonstrate the continuous dependence of the solution on the equation data, as such a property is always desirable because it implies small changes in the solution in response to small changes in the data. The framework within which these equations are studied is the hyperspace of subsets of an infinite-dimensional space of square-integrable random variables. This poses limitations, or rather challenges, in the numerical modeling of phenomena described by these equations. However, it opens the way for attempts to overcome this limitation in future research. The future studies may also address equations with even less stringent conditions imposed on the equation data than the current (A2) condition, as well as generalizations where the process governing stochastic fluctuations is a Lévy process, martingale, semimartingale or fractional Brownian motion.
